# The gut-liver-immune axis: structural basis, cellular architecture, molecular mediators, and disease-specific manifestations

**DOI:** 10.3389/fimmu.2026.1864805

**Published:** 2026-08-03

**Authors:** Sang Jun Yoon, Jung A Eom, Ki Tae Suk

**Affiliations:** Institute for Liver and Digestive Diseases, Hallym University College of Medicine, Chuncheon, Republic of Korea

**Keywords:** bile acids, gut-liver axis, gut-liver-immune axis, intestinal barrier, liver disease, macrophages, MAIT cells, Treg/Th17 balance

## Abstract

The gut and liver form an anatomical and functional unit linked by the portal circulation, the biliary tract, lymphatic drainage, and neuroimmune pathways. In recent years, the classical gut-liver axis has expanded from a predominantly microbial and metabolic concept into a broader framework in which immune cells are viewed as central integrators of inter-organ signaling. In health, balanced microbial sensing, intact mucosal and vascular barriers, regulated bile acid signaling, and specialized tissue-resident immune programs preserve tolerance at both intestinal and hepatic interfaces. In disease, dysbiosis, barrier failure, altered bile acid pools, endogenous hepatotoxic metabolites, and aberrant immune-cell trafficking remodel the hepatic immune niche and drive steatohepatitis, alcohol-associated liver injury, cholangiopathy, cirrhosis, acute decompensation, and hepatocellular carcinoma. This review reframes the gut-liver axis as a gut-liver-immune axis and synthesizes current evidence on its structural basis, cellular architecture, molecular mediators, and disease-specific manifestations. We discuss the roles of macrophages, dendritic cells, neutrophils, innate-like T cells, natural killer cells, and adaptive T-cell subsets in translating gut-derived signals into hepatic inflammation, tissue repair, fibrosis, or tumor surveillance. We also highlight the bidirectional role of the liver in shaping intestinal immunity, particularly through bile acids and hepatobiliary-derived mediators. Finally, we examine current therapeutic strategies targeting the microbiota, mucosal barrier, bile acid signaling, and immune-cell trafficking, and we outline major conceptual and translational gaps that should guide the next generation of mechanistic and clinical studies.

## 1 Introduction

Liver disease is a major and growing global health problem, and mechanistic models that integrate metabolism, barrier biology, inflammation, and host-microbe interactions are increasingly required to explain its heterogeneity across etiologies and stages (1, 2). The gut-liver axis is now understood as a bidirectional network rather than a simple one-way conduit through which microbial products reach the liver. The portal vein delivers gut-derived nutrients, metabolites, microbial molecules, and immune mediators to the liver, whereas bile acids (BA), immunoglobulins, antimicrobial programs, and hepatobiliary signals return from the liver to shape the intestinal ecosystem and mucosal immunity (3–7). This systems-level view has become especially relevant as studies in metabolic, alcohol-associated, cholestatic, cirrhotic, and malignant liver disease increasingly converge on shared mechanisms of dysbiosis, barrier disruption, altered metabolite production, and immune rewiring (8–13). Dysbiosis refers to an imbalance in the microbial community characterized by impaired diversity, loss of beneficial commensals, and/or expansion of potentially pathogenic taxa, which can compromise barrier integrity and host immune function. Barrier disruption, colloquially termed leaky gut, increased intestinal permeability denotes the pathological loss of tight-junction integrity and selective barrier function at the epithelial interface, permitting translocation of microbial products (e.g., lipopolysaccharide (LPS), flagellin, bacterial DNA) and dietary antigens into the portal and systemic circulation.

Historically, gut-liver research emphasized the composition of the microbiome, intestinal permeability, and endotoxin exposure. Those components remain central, but they do not fully explain why similar microbial or metabolic perturbations can produce distinct liver phenotypes, why intestinal disease can be associated with hepatobiliary inflammation at distant sites, or why therapeutic responses vary markedly between patients with the same clinical diagnosis. A major conceptual advance has been the recognition that immune cells translate these gut-derived cues into tissue-specific outcomes. Hepatic and intestinal macrophages, dendritic cells (DC), neutrophils, mucosal-associated invariant T (MAIT) cells, natural killer T (NKT) cells, natural killer (NK) cells, and T-cell subsets such as Th17 and regulatory T cells (Treg) do not simply react to inflammation after it is established; instead, they shape the trajectory from homeostasis to inflammation, fibrosis, failed repair, or tumor surveillance (8, 9, 14–18).

This review positions the immune system as the defining third node the ‘X’ of the gut-liver-X axis, uses the term gut-liver-immune axis, and argues that immune cells are the principal mechanism through which inter-organ communication acquires tissue specificity and pathological consequence. We first summarize the anatomical and physiological circuits that permit signaling between intestine and liver. We then discuss the immune architecture of the axis, with attention to how tissue residency, recruited populations, and immune-cell trafficking create a multilayered interface between luminal contents and hepatic responses. Next, we review the major molecular mediators that drive crosstalk, including microbe-associated molecular patterns (MAMP), BAs, short-chain fatty acids (SCFA), tryptophan metabolites, endogenous ethanol, and fungal toxins. Finally, we discuss disease-specific manifestations in metabolic dysfunction-associated steatotic liver disease (MASLD)/metabolic dysfunction-associated steatohepatitis (MASH), alcohol-associated liver disease (ALD), primary sclerosing cholangitis (PSC) and inflammatory bowel disease (IBD)-associated hepatobiliary disease, cirrhosis and acute decompensation (AD), and hepatocellular carcinoma (HCC), before closing with therapeutic opportunities and unresolved questions (3–6, 15).

This framing is intended to be more than terminological. Earlier gut-liver models, however sophisticated in their treatment of microbial composition, endotoxemia, or bile acid flux, generally cast immune cells as downstream effectors that respond once a microbial or metabolic perturbation has already been transmitted to the liver. We instead argue that immune cells are integrators in three specific and testable senses: first, they assign tissue-specific meaning to otherwise identical portal signals, so that the same LPS or BA concentration can yield tolerance, controlled defense, or fibrogenesis depending on the receiving cell and its niche; second, they are not stationary readouts but mobile disease vectors, since gut-imprinted lymphocytes can themselves relocate to and injure the liver; and third, they act bidirectionally, feeding back onto barrier integrity and microbial ecology rather than merely receiving signals. It is this combination of context-assignment, mobility, and feedback, rather than the mere presence of inflammation, that distinguishes the gut-liver-immune framework from the classical microbe → metabolite → hepatocyte paradigm (3–9, 14, 15).

## 2 Structural and functional basis of the gut-liver-immune axis

### 2.1 Anatomical circuits linking intestine and liver

The gut and liver are physically connected by several overlapping circuits. The portal circulation is the most familiar route, delivering roughly seventy percent of hepatic blood flow from the intestine and exposing the liver to absorbed nutrients, microbial antigens, metabolites, xenobiotics, and inflammatory mediators. Enterohepatic circulation provides a second route by which hepatocyte-derived BAs are secreted into bile, metabolized by intestinal microorganisms, and returned to the liver as both detergents and signaling molecules. Lymphatic drainage and neural pathways add further dimensions to this bidirectional network, making the gut-liver axis more accurately described as a multi-route inter-organ communication system (3, 5–7, 15). The neural component of this network is increasingly defined at a mechanistic level rather than invoked only in the abstract. The efferent vagus mediates a cholinergic anti-inflammatory pathway in which acetylcholine, acting on α7-nicotinic receptors expressed by macrophages, suppresses pro-inflammatory cytokine release; this reflex was first established for systemic endotoxin responses and is directly relevant to the Kupffer-cell–rich hepatic compartment (19). More specifically for the gut-liver interface, a defined gut–brain–liver neural arc has been shown to maintain the intestinal regulatory T-cell niche: hepatic vagal sensory input is integrated centrally and relayed back to the gut, where it controls peripheral Treg numbers, demonstrating that hepatic afferent signaling actively shapes mucosal immune tone (20). The immune tone denotes the basal set-point of immune reactivity within a tissue that the integrated threshold, established by resident immune cells, stromal cues, and local metabolites, that determines whether an incoming signal yields tolerance, controlled defense, or destructive inflammation. These circuits provide a non-humoral, rapid channel through which hepatic and intestinal immune states are coupled, complementing the slower metabolite- and trafficking-based mechanisms emphasized elsewhere in this review, and they suggest that neuroimmune modulation (e.g., vagal stimulation) merits consideration as an axis-directed strategy (3, 5–7, 15).

This anatomy helps explain why the liver functions as a second firewall after the intestinal barrier. It is important to distinguish this second firewall function from the related but mechanistically separate concept of hepatic immune tolerance. The firewall concept is fundamentally quantitative and containment-based: it refers to the liver’s capacity to physically capture, sequester, and clear commensal bacteria and their products that breach the intestinal barrier and enter the portal blood, thereby preventing systemic dissemination. This was demonstrated directly in experimental models showing that the liver intercepts and eliminates gut commensals reaching the systemic circulation, acting as a vascular firewall whose failure permits spillover and systemic immune activation (21). Hepatic immune tolerance, by contrast, is a qualitative property of the hepatic antigen-presenting environment, the bias of Kupffer cells, liver sinusoidal endothelial cells (LSECs), and hepatic DCs toward non-responsiveness or regulatory output when sampling portal antigen. The two are complementary: the firewall determines how much microbial material is cleared versus retained, whereas tolerance determines how the retained material is interpreted immunologically. Conflating them obscures the fact that disease can arise either from firewall failure (excess translocation overwhelming clearance) or from a breakdown of tolerance (pathological interpretation of normally tolerated signals), and that these two modes may require different therapeutic logics (3, 7, 22–24). Portal blood arriving from the gut is normally filtered by sinusoidal endothelial structures, Kupffer cells, and other resident immune populations that sample intestinally derived material without necessarily triggering destructive inflammation. At the same time, the liver influences the intestine through bile composition, secreted proteins, and the metabolic state imposed on the luminal and mucosal environment. BAs are particularly important because they influence epithelial integrity, microbial ecology, and immune differentiation in the gut while also feeding back on hepatic metabolism and inflammation (3, 4, 15, 25–27).

### 2.2 Barrier systems that maintain homeostasis

The homeostatic gut-liver-immune axis depends on nested barriers. At the luminal interface, mucus and mucins physically separate microbiota from the epithelium, whereas antimicrobial peptides and immunoglobulin A (IgA) restrict microbial encroachment and maintain spatial segregation between host tissues and microbial communities (28–31). The epithelial layer itself forms a selective barrier using tight junctions and specialized transport programs, while the gut vascular barrier (GVB) controls access of luminal molecules and microbes to the portal circulation. These two structures are serially arranged and functionally non-redundant, and they should not be treated as a single gut barrier. The epithelial barrier is the most luminal layer: a single cell sheet whose paracellular permeability is governed by tight-junction complexes (claudins, occludin, ZO-1) and whose integrity controls the initial flux of luminal antigens and small molecules. The GVB lies deeper, at the level of the intestinal microvasculature, and is composed of endothelial cells supported by pericytes and enteric glial cells; it constitutes a second, endothelial checkpoint that specifically restricts the entry of bacteria and larger microbial products into the portal blood even after the epithelium has been crossed. The GVB was identified as a discrete structure analogous to the blood–brain barrier and was shown to limit systemic bacterial dissemination, with its disruption (e.g., via PV1/PLVAP upregulation) permitting translocation that the epithelium alone cannot prevent (32). This distinction is mechanistically consequential: epithelial tight-junction loss and GVB failure can occur independently, produce different translocation spectra (small soluble antigens vs. intact bacteria), and may require different barrier-directed interventions, an important nuance given that leaky gut is frequently invoked without specifying which barrier has failed (3, 7, 22, 33). On the hepatic side, LSECs, Kupffer cells, and zonated immune surveillance systems limit the dissemination of gut-derived material and preserve tolerance under basal conditions (3, 7, 22–24).

Homeostasis within these barriers is not immunological silence. Rather, it is an actively maintained equilibrium in which controlled microbial sensing educates epithelial cells, stromal cells, and immune cells. Commensal bacteria shape nutrient gradients, mucus properties, epithelial metabolism, and antimicrobial programming, thereby influencing the inflammatory threshold of the intestine and the liver. Short-chain fatty acids and other microbial metabolites condition macrophages and regulatory lymphocytes toward tolerance-promoting phenotypes, while BA receptor signaling constrains bacterial overgrowth and translocation. The resulting balance allows the host to tolerate constant exposure to microbial products while remaining prepared for pathogen invasion (34–39).

### 2.3 Microbiota composition and the multi-kingdom intestinal ecosystem

The intestinal ecosystem relevant to liver disease extends beyond bacteria. Metagenomic studies established the scale and diversity of the gut microbiome, while later work showed that the gut virome and mycobiome are dynamic components of host physiology and disease susceptibility (40–44). In liver disease, changes in viral and fungal communities are not merely epiphenomena. Studies in MASLD and alcohol-associated liver disease have linked alterations in the virome or mycobiome to fibrosis severity, alcoholic hepatitis, and systemic anti-fungal immune responses, suggesting that the gut-liver-immune axis should be understood as a multi-kingdom ecological system (45–49). Beyond the parallel existence of bacterial, viral, and fungal communities, their interactions across kingdoms carry distinct immunological consequences that a single-kingdom view cannot capture. Commensal bacteria normally constrain fungal expansion through competition and metabolite production, so that bacterial depletion, whether from antibiotics, alcohol, or dysbiosis, can license overgrowth of pathobiont fungi such as *Candida albicans* and the consequent release of effectors like candidalysin, linking a bacterial perturbation to a fungal-driven, type-17–biased hepatic inflammatory response (50). Bacteriophages, in turn, regulate bacterial community structure and can be harnessed to deplete specific bacterial pathobionts (e.g., cytolysin-positive *Enterococcus faecalis*), illustrating viral-on-bacterial control with direct hepatic consequences. These trans-kingdom dependencies explain why compositional profiling of any single kingdom in isolation is often insufficient, and why multi-kingdom, function-centered analyses are required to predict the net immunological output reaching the liver (4, 47, 48, 51–55). It should be noted, however, that for most viral and fungal signals the evidence currently remains at the level of association, and mechanistic causality meeting the full criteria for a disease driver has been established for only a subset of these communities; the field should therefore interpret composition findings alongside functional and host-immune data rather than equating association with causality.

Microbial composition also influences hepatoprotection. Commensal microbiota can prevent fibrosis and shape zonated hepatic immunity, whereas dysbiotic communities can transfer susceptibility to fatty liver disease or alcohol-associated injury in preclinical models (23, 56–58). These findings are important because they show that the microbiome is not only associated with liver disease but can also determine disease transmissibility and tissue phenotype. Even so, taxonomic composition alone is insufficient; the biological consequence of dysbiosis depends on the functional metabolites and immune programs associated with a given community structure (4, 5, 59).

### 2.4 Bidirectionality: how the liver shapes intestinal immunity

Recent work has expanded the axis from a gut-to-liver model to a truly bidirectional network. The liver continuously shapes intestinal immunity through BA synthesis and transport, complement-related programs, metabolic signaling, and hepatobiliary effects on microbial ecology (3, 15, 25). BAs are especially central because primary BAs synthesized in the liver are transformed by the microbiota into secondary BAs that act through farnesoid X receptor (FXR), Takeda G-protein coupled receptor 5 (TGR5), and related receptors to regulate epithelial integrity, antimicrobial function, and immune-cell differentiation. Disturbance of this loop can produce intestinal bacterial overgrowth, GVB dysfunction, altered T-cell polarization, and feed-forward hepatic inflammation (22, 26, 27, 39, 60).

**Figure 1:**
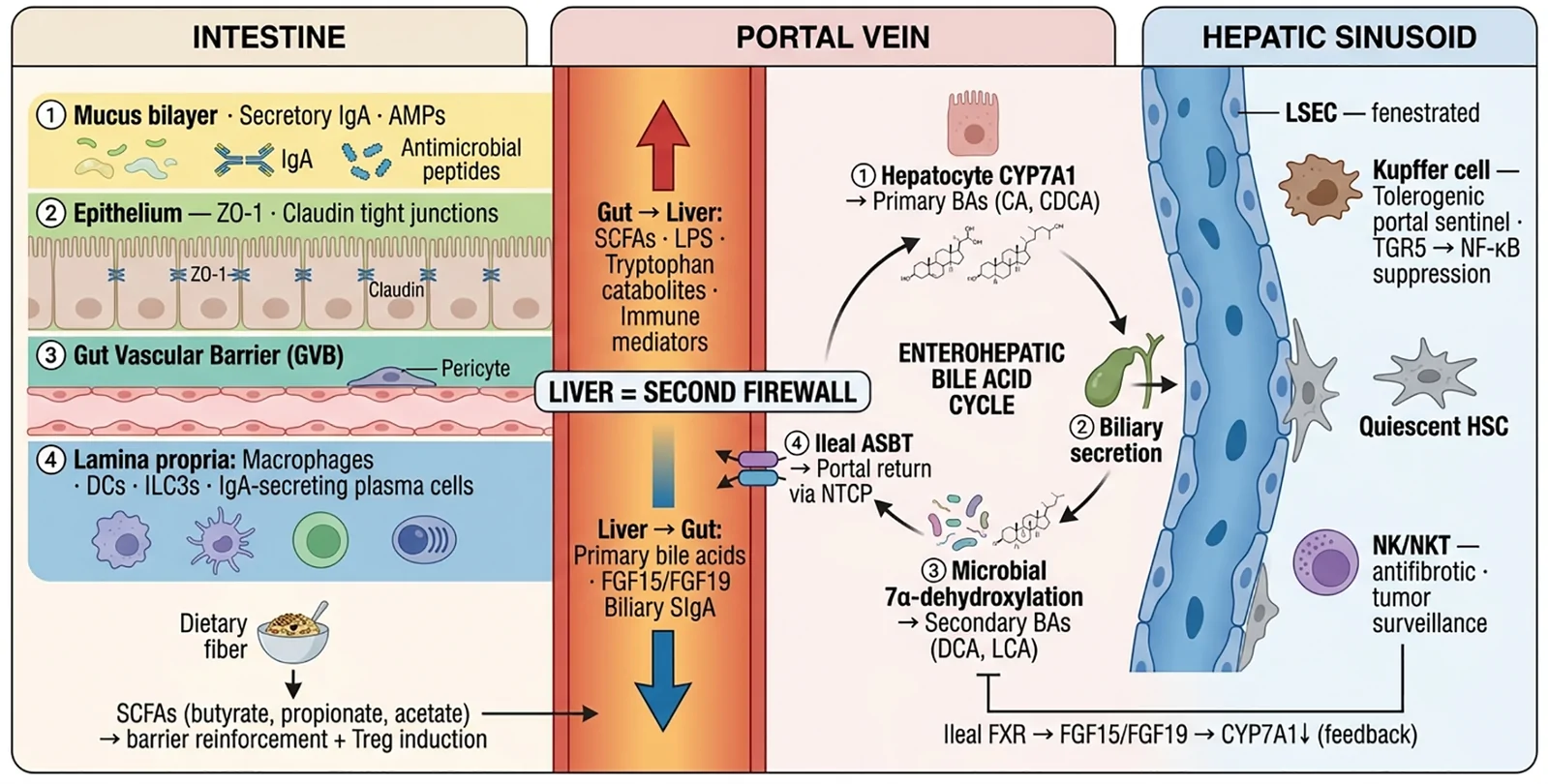
Structural and physiological overview of the gut-liver-immune axis in health. The bidirectional anatomical framework connecting the intestine and liver across three anatomical sections. Left section (Intestine): the intestinal wall is depicted in cross-section showing four color-banded, numbered barrier layers arranged from lumen to submucosa (1): mucus bilayer with secretory IgA and antimicrobial peptides (2); tight-junction-sealed epithelium (ZO-1, claudins) (3); subepithelial GVB; and (4) lamina propria containing macrophages, DCs, ILC3s, and IgA-secreting plasma cells; a side panel notes that commensal fermentation of dietary fiber produces SCFAs (butyrate, propionate, acetate) that reinforce barrier integrity and promote Treg induction. Center section (Portal vein and enterohepatic cycle): the portal vein is shown as the primary afferent conduit with a bold red upward arrow labeled with gut-to-liver signals (SCFAs, LPS, tryptophan catabolites, immune mediators) and a bold blue downward arrow labeled with liver-to-gut signals (primary BAs, FGF15/FGF19, biliary SIgA); adjacent to the portal vessel, the enterohepatic BA cycle is depicted as a four-step clockwise loop encompassing hepatocyte CYP7A1-driven primary BA synthesis (CA, CDCA), biliary secretion, microbial 7α-dehydroxylation to secondary BAs (DCA, LCA), and ileal ASBT-mediated reabsorption with hepatocyte NTCP recapture; a central banner designates the liver as the “Second Firewall.” Right section (Hepatic sinusoid): the sinusoidal niche depicts fenestrated LSECs and Kupffer cells maintaining tolerogenic portal immune surveillance, with Kupffer-cell TGR5 activation suppressing NF-κB-mediated inflammation (cAMP-PKA pathway), NK/NKT cells in antifibrotic and tumor-surveillance positions, and quiescent HSCs in the perisinusoidal space of Disse; an inset arrow illustrates the ileal FXR → FGF15/FGF19 → hepatic CYP7A1 negative-feedback loop that couples intestinal BA sensing to hepatic synthesis rate. Color-coded bidirectional arrows distinguish gut-to-liver (red) from liver-to-gut (blue) communication pathways. AMP, antimicrobial peptide; ASBT, apical sodium-dependent bile acid transporter; BA, bile acid; CA, cholic acid; cAMP, cyclic adenosine monophosphate; CDCA, chenodeoxycholic acid; CYP7A1, Cholesterol 7α-Hydroxylase; DCA, deoxycholic acid; FGF15/FGF19, fibroblast growth factor 15 (rodent)/19 (human); FXR, farnesoid X receptor; GVB, gut vascular barrier; HSC, hepatic stellate cell; IgA, immunoglobulin A; ILC3, group 3 innate lymphoid cell; LCA, lithocholic acid; LPS, lipopolysaccharide; LSEC, liver sinusoidal endothelial cell; NF-κB, nuclear factor kappa B; NK, natural killer; NKT, natural killer T cell; NTCP, sodium-taurocholate co-transporting polypeptide; PKA, protein kinase A; SCFA, short-chain fatty acid; SIgA, secretory immunoglobulin A; TGR5, Takeda G-protein coupled receptor 5; Treg, regulatory T cell; ZO, zonular occludens.

This reverse directionality matters conceptually and clinically. It helps explain why cholestasis, cirrhosis, and altered BA pools can worsen intestinal permeability and dysbiosis; why liver disease can generate systemic and mucosal immune defects; and why intestinal immune homeostasis may be restored or worsened by hepatic interventions. In this sense, the liver is not simply the recipient of intestinal signals but a determinant of the immunological context in which those signals are interpreted (15, 25, 33, 61, 62) (**Figure 1**).

## 3 Immune architecture of the gut-liver-immune axis

### 3.1 Macrophages as sentinel cells at both ends of the axis

Macrophages are among the most important cell types in the gut-liver-immune axis because they occupy strategic niches in both intestine and liver and display extraordinary functional plasticity. In the intestine, resident macrophages help clear apoptotic material, support epithelial turnover, and tolerate commensal-derived signals while remaining capable of responding to invasive organisms. In the liver, Kupffer cells are continuously exposed to portal blood and act as tolerogenic sentinels that capture microbial products and shape hepatic immune tone. Chronic injury, however, recruits monocyte-derived macrophages that differ from homeostatic Kupffer cells in phenotype and function and often promote inflammatory and fibrogenic circuits (6–9).

Macrophage phenotypes are strongly influenced by microbial metabolites and barrier status. Butyrate can imprint antimicrobial functions in macrophages, whereas dysbiosis, endotoxin exposure, and altered BA signaling promote a switch toward inflammatory activation. These responses extend beyond cytokine production. Macrophages regulate chemokine gradients, endothelial activation, stellate cell behavior, and antigen presentation, placing them at the intersection of microbial sensing and tissue remodeling (25, 36, 56, 57, 63). For this reason, macrophages can connect events such as intestinal barrier disruption or changes in BA composition to downstream fibrosis, cholangiopathy, or tumor-promoting inflammation.

The notion that macrophages form a shared intestinal-hepatic network is increasingly supported by spatial and niche-based studies. The intestinal macrophage compartment contains subsets associated with epithelium, blood vessels, neurons, and Peyer’s patches, whereas the liver contains distinct zonated myeloid populations that integrate portal signals and local tissue cues. These niches create a context in which gut-derived signals do not have a single uniform outcome. Instead, location, local metabolites, and disease stage determine whether macrophage responses support tolerance, antimicrobial defense, repair, fibrosis, or cancer-promoting immunoregulation (9, 15, 23).

### 3.2 DCs and antigen presentation across tissues

DCs provide a mechanistic bridge between luminal content and adaptive immunity. In the intestine, they sample antigens, migrate to lymphoid structures, and promote either tolerance or inflammatory T-cell differentiation depending on context. In the liver, DCs reside in a classically tolerogenic environment but can become activated during injury and contribute to inflammatory amplification. Because liver disease often alters both antigen flux and cytokine milieu, DC behavior is reshaped as the gut-liver axis moves from homeostasis to chronic disease (6, 8, 14).

The antigenic repertoire handled by DCs in this setting is unusually diverse, including microbial molecules, food-derived antigens, endogenous lipids, and BA-modulated signals. These features are relevant to both metabolic and autoimmune liver disease because they help determine whether T-cell responses remain regulated or become pathogenic. The gut-liver-immune axis thus should not be viewed merely as an exposure problem but as a continuous antigen-processing problem in which DCs and other antigen-presenting cells define the quality of downstream immune responses (3, 4, 8).

### 3.3 Neutrophils and acute inflammatory amplification

Neutrophils are rapid responders to barrier disruption, fungal toxins, bacterial translocation, and acute inflammatory exacerbations. In alcohol-associated hepatitis and advanced cirrhosis, neutrophils are prominent participants in inflammatory injury, yet they also exhibit paradoxical dysfunction that may contribute to infection susceptibility. This dual phenotype is characteristic of the broader immune paradox of advanced liver disease, in which hyperinflammation and immune paralysis coexist (10, 13, 62, 64).

Signals originating in the intestine, including candidalysin, microbial endotoxin, and altered metabolites, can directly or indirectly enhance neutrophil recruitment and activation. Studies of fungal toxins and epithelial type 17 responses suggest that the mycobiome is a particularly relevant driver of neutrophil-rich inflammation in some settings. As a result, neutrophils are not only markers of severe disease but also effectors through which gut-derived danger signals reshape hepatic injury (47, 51–54).

### 3.4 Innate-like lymphocytes: MAIT cells, NKT cells, and NK cells

MAIT cells provide one of the clearest examples of immune cells acting as inter-organ sensors. They are enriched in the liver, recognize microbial riboflavin-derived metabolites presented by MHC-related protein 1 (MR1), and can also be activated by cytokines such as interleukin (IL)-12 and IL-18. Because their canonical ligands are microbial in origin and portal delivery exposes the liver to these products, MAIT cells are ideally positioned to translate changes in microbiota composition or barrier function into hepatic immune outputs (16, 17, 65). In chronic liver disease, MAIT cell numbers and functions are often altered, but their roles appear context dependent: they may become promote antibacterial defense or contribute to fibrosis and inflammatory cytokine production (18, 66–68). In MASLD/MASH, microbiota-activated MAIT cells have been linked to intestinal permeability and inflammatory signaling, suggesting that they sit at the interface between microbial metabolism, mucosal immune dysfunction, and hepatic inflammation (17, 69). These observations fit a broader model in which innate-like T cells act less as static disease markers and more as dynamic interpreters of microbial metabolite tone. They may therefore represent useful biomarkers of axis activity as well as targets for mechanistic studies.

NKT cells are another critical lymphocyte population within the hepatic immune niche. Their relevance to the gut-liver-immune axis became especially clear when microbiota-dependent BA metabolism was shown to regulate hepatic chemokine expression and NKT-cell accumulation, thereby influencing liver cancer control. This framework strongly supports the idea that microbial communities, BAs, endothelial programs, and hepatic immune surveillance are functionally coupled (5, 25, 70). NK cells participate in antifibrotic and antitumor defense but can be impaired or reprogrammed by chronic inflammatory and metabolic conditions; changes in cytokine milieu, BA signaling, and microbial products clearly affect their function in chronic liver disease and HCC (14, 64, 71).

### 3.5 Adaptive immunity and the Th17-Treg balance

Adaptive immune polarization is a central downstream effect of the gut-liver-immune axis. The balance between Th17 cells and Treg is particularly relevant because it links mucosal perturbation to systemic inflammation, epithelial barrier defense, tolerance, and fibrosis-related immune tone. Microbial metabolites and BA derivatives can directly alter this balance. Commensal-derived short-chain fatty acids promote regulatory pathways, whereas several BA metabolites have been shown to enhance Treg generation or suppress Th17 differentiation (37, 72–74). Dysbiosis does not simply increase antigen burden; it changes the repertoire of immunoregulatory molecules to which intestinal and hepatic immune cells are exposed. In metabolic liver disease, altered BA pools and tryptophan metabolites are associated with disrupted tolerance and ongoing inflammation. In alcohol-associated disease and fungal dysbiosis, similar immune shifts may occur through different mediators, including IL-17-associated responses and barrier-dependent cytokine cascades (53, 75–78). The clinical phenotype therefore reflects both the nature of the trigger and the local immune ecosystem in which it is interpreted (5, 14, 15).

### 3.6 Immune-cell trafficking as an inter-organ mechanism

The phrase inter-organ dynamics is most concrete when immune-cell trafficking is directly demonstrated. PSC and IBD-associated hepatobiliary disease provide the clearest human example. Studies in patients have shown that gut-primed lymphocytes can home to the liver, and that aberrant expression of gut-homing chemokine pathways such as C-C chemokine ligand (CCL)25 – C-C chemokine receptor (CCR)9 and integrin-associated mechanisms can recruit mucosal lymphocytes to inflamed hepatic tissue (79, 80). These observations established an enterohepatic recirculation model in which cells educated in the gut may later participate in liver inflammation independent of overt intestinal activity.

**Figure 2:**
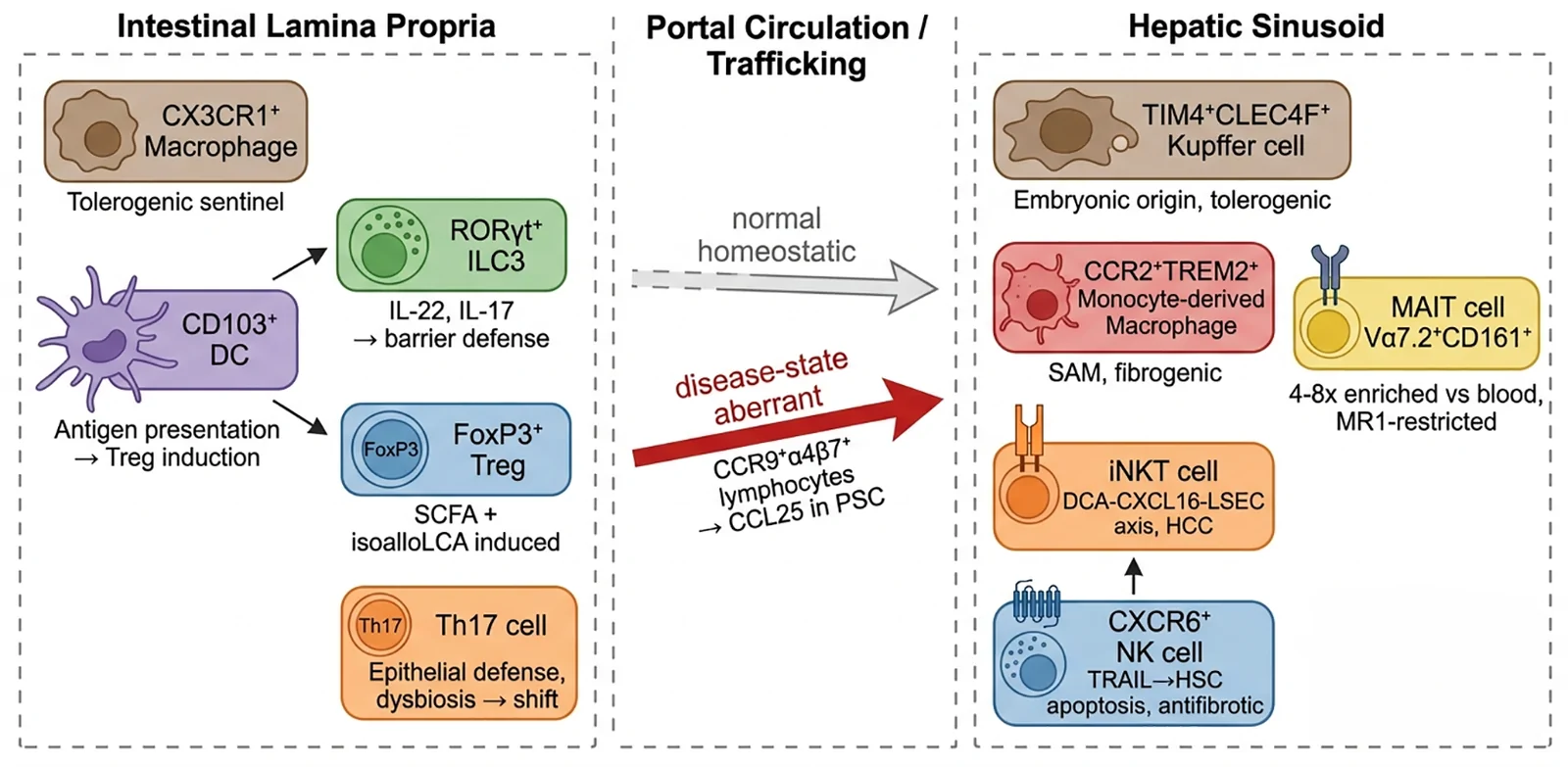
Immune-cell atlas of the gut-liver-immune axis: tissue residency, trafficking, and context-dependent function. Maps major immune populations across three anatomical compartments separated by dashed vertical borders. In the intestinal lamina propria: CX3CR1^+^ macrophages (tolerogenic sentinels), CD103^+^ DCs (Treg-inducing antigen presentation), RORγt^+^ ILC3s (IL-22/IL-17 production), FoxP3^+^ Tregs (sustained by SCFAs and isoalloLCA), and Th17 cells (epithelial defense; shift toward inflammatory phenotype in dysbiosis). In the portal circulation and trafficking zone: homeostatic (thin gray arrows) versus disease-state aberrant trafficking (bold red arrow) showing CCR9^+^α4β7^+^ lymphocytes aberrantly recruited to hepatic portal tracts via CCL25 in PSC. In the hepatic sinusoid: TIM4^+^CLEC4F^+^ Kupffer cells (embryonic, tolerogenic), CCR2^+^TREM2^+^ monocyte-derived macrophages (scar-associated, fibrogenic), MAIT cells (Vα7.2^+^CD161^+^, MR1-restricted, 4–8-fold liver-enriched vs. blood), iNKT cells (accumulated via the DCA-CXCL16-LSEC axis, context-dependent role in HCC), and CXCR6^+^ NK cells (antifibrotic TRAIL-mediated HSC apoptosis). CCL, C-C chemokine ligand; CCR, C-C chemokine receptor; CD, cluster of differentiation; CLEC4F, C-type lectin domain family 4 member F; CXCL, C-X-C chemokine ligand; CXCR, C-X-C chemokine receptor; CX3CR1, CX3C motif chemokine receptor 1; DC, dendritic cell; DCA, deoxycholic acid; FoxP3, Forkhead box P3; HCC, hepatocellular carcinoma; HSC, hepatic stellate cell; IL, interleukin; ILC3, group 3 innate lymphoid cell; iNKT, invariant natural killer T cell; isoalloLCA, isoallolithocholic acid; LSEC, liver sinusoidal endothelial cell; MAIT, mucosal-associated invariant T cell; MR1, MHC-related protein 1; NK, natural killer; PSC, primary sclerosing cholangitis; RORγt, RAR-related orphan receptor gamma t; SAM, scar-associated macrophage; SCFA, short-chain fatty acid; TIM4, T cell membrane protein 4; TRAIL, TNF-related apoptosis-inducing ligand; Treg, regulatory T cell; TREM2, triggering receptor expressed on myeloid cells 2.

In PSC, analysis of gut-homing molecules in non-endstage disease further supports the idea that aberrant lymphocyte localization is a disease-defining process rather than a late by-product of advanced fibrosis (12, 14, 81, 82). Trafficking programs are not uniform across the disease course; they reconfigure as injury progresses from an inflammatory through a fibrotic to an oncogenic stage. In the early inflammatory stage, recruitment is dominated by CCR2+ monocyte-derived macrophages responding to CCL2 and by CCR9+α4β7+ gut-imprinted lymphocytes homing via endothelial CCL25, as exemplified in PSC-IBD. As injury becomes fibrotic, single-cell analyses of human cirrhosis have identified a discrete population of TREM2+CD9+ scar-associated macrophages that expand within the fibrotic niche and occupy a profibrogenic, spatially restricted compartment distinct from homeostatic Kupffer cells, indicating that the dominant trafficking and differentiation program shifts toward scar-associated myeloid states (83). In the oncogenic stage, the relevant axes change again: microbiota-BA-dependent C-X-C chemokine ligand (CXCL)16 expression by liver sinusoidal endothelial cells governs hepatic NKT-cell accumulation and tumor surveillance, while the tumor microenvironment is increasingly characterized by exhausted CD8+ T cells and immunosuppressive tumor-associated macrophages. Recognizing these stage-specific programs is therapeutically important because a trafficking-directed intervention rational in the inflammatory stage (e.g., anti-α4β7 or anti-CCR9) may be ineffective or even counterproductive once fibrotic or tumor-promoting niches are established (12, 14, 70, 79–82). Similar concepts are increasingly invoked in checkpoint hepatitis, autoimmune liver disease, and cirrhosis-associated systemic immune dysregulation, although direct evidence varies by disease and cell type. (**Figure 2**, **Table 1**)

**Table 1:** Major immune-cell populations in the gut-liver-immune axis and their roles in homeostasis and disease.

Cell type	Primary location(s)	Key markers/phenotype	Role in homeostasis	Role in disease/dysfunction	Gut-liver-immune axis relevance	Key refs
Kupffer cells (tissue-resident macrophages)	Hepatic sinusoids	CD68^+^, TIM4^+^, CLEC4F^+^; embryonic origin	Tolerogenic sentinel; scavenges portal LPS, apoptotic debris; maintains hepatic tolerance	Activates upon chronic portal LPS/dysbiosis exposure; promotes TNF-α, IL-6, TGF-β; fibrogenic co-activator of stellate cells	First-line hepatic filter of gut-derived microbial products; phenotype conditioned by BA and metabolite milieu	(6–9)
Monocyte-derived macrophages	Recruited to liver during injury; intestinal lamina propria	CD68^+^, CD14^+^, CCR2^+^; bone-marrow origin	Wound repair; supports mucosal restitution under controlled conditions	Promotes inflammatory fibrogenesis; contributes to profibrotic M2-like and scar-associated macrophage states	Amplifies gut-to-liver inflammatory signal; dysbiosis and barrier failure increase monocyte recruitment	(8, 9, 23)
Intestinal macrophages (lamina propria)	Small intestinal and colonic lamina propria; perivascular, subepithelial, myenteric niches	CD68^+^, CX3CR1^+^, CD163^+^; anti-inflammatory phenotype	Tolerates commensal signals; supports epithelial turnover, clears apoptotic cells; maintains mucosal homeostasis	Dysbiosis shifts toward inflammatory activation; contributes to barrier disruption and cytokine-driven epithelial injury	Upstream sentinel of the axis; altered phenotype translates dysbiosis into increased barrier permeability	(9, 15)
DCs	Intestinal lamina propria, mesenteric lymph nodes, hepatic sinusoids and portal tracts	CD11c^+^, MHC II^+^; plasmacytoid (pDC) and conventional (cDC1/cDC2) subsets	Antigen sampling; tolerogenic DC programs promote IgA class-switch and Treg induction	Activated DCs promote Th1/Th17 polarization; contribute to autoimmune and metabolic hepatic inflammation	Bridge between luminal antigen flux and adaptive immune output; hepatic DCs interpret portal antigen context	(6, 8, 14)
Neutrophils	Portal blood, hepatic sinusoids (recruited); intestinal epithelial interface during inflammation	CD66b^+^, MPO^+^, CXCR2^+^	Rapid antimicrobial defense; first responders to barrier breach	Dual dysfunction in ALD/cirrhosis: hyperinflammatory damage AND impaired killing; candidalysin/LPS-driven recruitment; neutrophil extracellular traps (NETs) amplify hepatic injury	Effectors of gut-derived danger signals (LPS, candidalysin, microbial toxins) in the liver; marker and driver of AD	(10, 47, 51–54, 62–64)
MAIT cells	Liver (highly enriched), intestinal mucosa, peripheral blood	Vα7.2^+^, CD161^+^, MR1-restricted; CD8^+^ or DN phenotype	Antibacterial surveillance via microbial riboflavin (5-OP-RU) presented by MR1; also responsive to IL-12/IL-18	Depleted from blood in chronic liver disease; tissue accumulation with altered function; context-dependent: antibacterial OR profibrotic/inflammatory	Direct sensors of gut microbiota-derived metabolites; portal delivery links intestinal ecology to hepatic MAIT-cell activation	(16–18, 65–69)
NKT cells	Liver (highly enriched), intestinal lamina propria	CD3^+^, NK1.1^+^/CD56^+^; invariant Vα14-Jα18 (iNKT); CD1d-restricted	Lipid antigen surveillance; iNKT cells link microbial lipids to rapid IFN-γ/IL-4 production; tumor immune surveillance	Accumulation in liver driven by microbiota-dependent BA and CXCL16 axis; promote hepatic fibrosis and HCC in some contexts	Key effectors of microbiota–BA–endothelial–immune surveillance circuit; control NKT accumulation determines HCC risk	(5, 25, 70)
NK cells	Liver (tissue-resident and circulating), intestinal epithelium (IEL subset)	CD56^+^, CD3^−^, NKp46^+^; liver-resident: CXCR6^+^	Antifibrotic via TRAIL-mediated HSC killing; antitumor cytotoxicity; IFN-γ-mediated antiviral defense	Impaired cytotoxicity in cirrhosis, ALD, and HCC; reprogrammed by chronic inflammatory cytokines, BAs, and hypoxia	Antifibrotic and antitumor programs are shaped by gut-derived metabolites, BA signaling, and chronic inflammatory milieu	(14, 64, 71)
Th17 cells	Intestinal lamina propria, liver portal tracts (in disease)	CD4^+^, RORγt^+^, IL-17A/F^+^, IL-22^+^	Epithelial barrier defense; antimicrobial via IL-17/IL-22-stimulated AMPs; mucosal homeostasis	Excessive Th17 in dysbiosis/ALD/MASH drives barrier-damaging IL-17; IL-17 promotes HSC activation and fibrosis	Th17 induction by dysbiosis and fungal/candidalysin signals links intestinal immune skewing to hepatic inflammatory progression	(53, 72–78)
Tregs	Intestinal lamina propria, mesenteric lymph nodes, liver	CD4^+^, CD25^+^, FoxP3^+^; IL-10^+^, TGF-β^+^	Maintains peripheral tolerance; suppresses excessive mucosal inflammation; shaped by SCFA and BA metabolites	Impaired Treg induction in dysbiosis (loss of SCFA, loss of Treg-inducing BA metabolites); inadequate Treg restraint drives chronic hepatic inflammation	Central mediators of microbiota-immune homeostasis; BA metabolites (UDCA, isoallolithocholic acid) promote hepatic and intestinal Treg differentiation	(37, 72–74)
Gut-primed mucosal lymphocytes (enterohepatic trafficking)	Intestine (origin); liver portal tracts (ectopic homing in PSC)	CCR9^+^, α4β7^+^, gut-educated imprinting markers	Intestinal immune surveillance; normal recirculation confined to gut-associated lymphoid tissue	Aberrant hepatic homing in PSC/IBD; CCL25-CCR9-mediated recruitment to hepatic endothelium drives portal and biliary inflammation	Exemplar of immune-cell trafficking as a gut-liver inter-organ disease mechanism	(11, 12, 79–82)

ALD, alcohol-associated liver disease; AMP, antimicrobial peptide; BA, bile acid; CCL, C-C chemokine ligand; CCR, C-C chemokine receptor; CD, cluster of differentiation; CLEC4F, C-type lectin domain family 4 member F; CXCL, C-X-C chemokine ligand; CXCR, C-X-C chemokine receptor; CX3CR1, CX3C motif chemokine receptor 1; DC, dendritic cell; DN, double negative; FoxP3, Forkhead box P3; HCC, hepatocellular carcinoma; HSC, hepatic stellate cell; IBD, inflammatory bowel disease; IEL, intraepithelial lymphocyte; IFN-γ, interferon-gamma; IgA, immunoglobulin A; IL, interleukin; iNKT, invariant natural killer T cell; LPS, lipopolysaccharide; MAIT, mucosal-associated invariant T cell; MASH, metabolic dysfunction-associated steatohepatitis; MPO, myeloperoxidase; MR1, MHC-related protein 1; NET, neutrophil extracellular trap; NK, natural killer; NKT, natural killer T cell; PSC, primary sclerosing cholangitis; RORγt, RAR-related orphan receptor gamma t; SCFA, short-chain fatty acid; TGF-β, transforming growth factor beta; TIM4, T cell membrane protein 4; TNF-α, tumor necrosis factor alpha; TRAIL, TNF-related apoptosis-inducing ligand; Treg, regulatory T cell; UDCA, ursodeoxycholic acid.

## 4 Molecular mediators driving gut-liver-immune crosstalk

### 4.1 Dysbiosis and barrier failure

Dysbiosis becomes pathogenic when it alters both microbial composition and the physical or functional integrity of host barriers. Tight-junction defects, mucus disruption, altered antimicrobial peptides, and GVB failure permit translocation of microbial products that normally remain compartmentalized. Experimental models show that barrier disruption can precede steatohepatitis, while clinical studies indicate that hepatic injury can in turn worsen intestinal permeability, reinforcing a vicious cycle (22, 84–86).

The concept of bacterial translocation should be expanded to include a spectrum of barrier-derived pathologies: movement of intact bacteria, exposure to microbial fragments such as lipopolysaccharide (LPS) or flagellin, flux of endogenous alcohol or amine metabolites, and passage of fungal toxins or other pathogen-associated molecular patterns (PAMP). In chronic liver disease and cirrhosis, barrier dysfunction is associated with small intestinal bacterial overgrowth, endotoxemia, altered BA profiles, and immune dysfunction; in MASLD and ALD it is tied more directly to inflammatory progression and disease severity (4, 13, 62, 87, 88).

### 4.2 Microbial products and pattern-recognition signaling

#### 4.2.1 Toll-like receptor signaling and inflammasome pathway

Microbial products activate hepatic and intestinal pattern-recognition systems, including toll-like receptors (TLRs), inflammasome pathways, and related sensors. These signaling pathways orchestrate cytokine release, endothelial activation, leukocyte recruitment, and downstream activation of stellate cells, thereby linking translocation to fibrosis. The importance of these pathways is evident in work showing that inflammasome-mediated dysbiosis alters MASLD progression and that TLR4-dependent responses to microbiota promote liver tumorigenesis (8, 57, 89, 90).

#### 4.2.2 Cell-type-specific sensor repertories and signal integration

Kupffer cells, monocyte-derived macrophages, DCs, neutrophils, epithelial cells, and hepatic stellate cells (HSC) all express overlapping but distinct repertoires of innate sensors. Thus, the same portal signal can produce different biological outputs depending on which cell type receives it, what metabolites are simultaneously present, and whether the tissue environment is tolerogenic, regenerative, fibrogenic, or tumor promoting (3, 8, 9).

### 4.3 BA signaling as a metabolic and immune hub

#### 4.3.1 BA synthesis, microbial transformation, and receptor systems

BAs are arguably the most characteristic molecular language of the gut-liver axis. Their synthesis in the liver, microbial transformation in the intestine, and receptor-mediated actions in both tissues make them uniquely suited to coordinate metabolism and immunity. FXR and TGR5 signaling influence epithelial defense, BA synthesis, microbial ecology, macrophage activation, cytokine production, and lymphocyte differentiation. Consequently, altered BA pools are relevant not only to cholestatic disease but also to MASLD, ALD, cirrhosis, and HCC (25–27, 61, 63, 76).

#### 4.3.2 BA dysregulation across disease states and BA-mediated immunoregulation

Human and animal studies demonstrate that BA dysregulation is present in MASH, alcoholic hepatitis, and cirrhosis, and that manipulating BA receptor pathways can modify disease phenotypes (63, 77, 91, 92). In addition, BA metabolites are now recognized as active immunoregulatory molecules that can shape the Treg-Th17 axis and thereby modulate tolerance at the gut-liver interface (72–74).

### 4.4 Metabolites beyond BAs: short-chain fatty acids, tryptophan, and endogenous ethanol

Short-chain fatty acids reinforce epithelial integrity, affect macrophage antimicrobial programming, and participate in systemic metabolic regulation (36, 37, 93). Tryptophan metabolism influences aryl hydrocarbon receptor (AHR) signaling, intestinal immune homeostasis, and alcohol-induced liver injury, providing a strong example of how microbial metabolism intersects with immunity rather than metabolism alone (75, 78). Indole and related compounds can alleviate steatosis and inflammatory signaling in experimental systems, supporting the idea that some microbial metabolites are protective and potentially therapeutically actionable (94, 95).

Conversely, endogenous or microbially derived hepatotoxic metabolites can intensify injury. Patients with MASLD or MASH may have higher portal or systemic exposure to endogenous ethanol or ethanol-producing microbes, and similar concepts extend to trimethylamine and other microbially modified molecules associated with alcohol-associated hepatitis or steatosis (96–99).

The mechanistic significance of these pathways lies in the fact that they reshape both epithelial vulnerability and hepatic immune signaling. Thus, metabolite-centered models are essential for understanding why dysbiosis affects disease progression differently across individuals.

**Figure 3:**
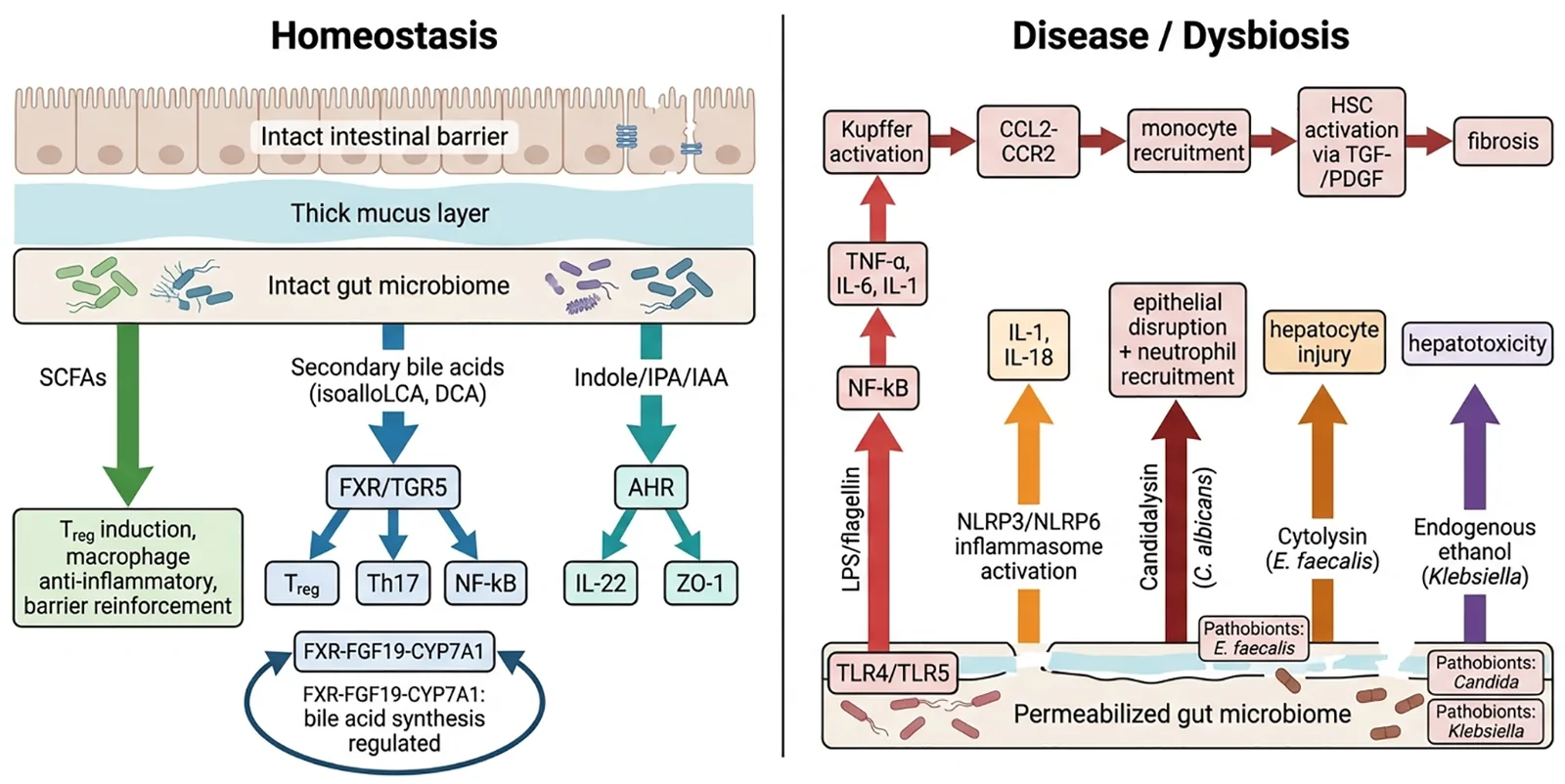
Molecular mechanisms driving disease along the gut-liver-immune axis. Contrasts the homeostatic state (left panel) with the pathological cascade initiated by dysbiosis and barrier disruption (right panel). Under homeostasis, commensal-derived SCFAs promote Treg induction and anti-inflammatory macrophage programming; secondary BAs activate FXR and TGR5; and indole/IPA/IAA engage the AHR pathway, all maintained within the ileal FXR–FGF15/FGF19–hepatic CYP7A1 feedback loop. In disease, expansion of Proteobacteria, *Candida*, and high-alcohol-producing *Klebsiella* depletes SCFAs and releases pathobiont toxins (candidalysin from *C. albicans*; cytolysin from *E. faecalis*), degrading tight-junction proteins, thinning the mucus layer, and permeabilizing the GVB. The resultant portal flood of LPS, flagellin, and microbial DNA activates hepatic TLR4, TLR5, and TLR9, triggers NLRP3/NLRP6 inflammasomes, and drives NF-κB-dependent production of TNF-α, IL-6, and IL-1β. Downstream effects include CCL2-CCR2-mediated monocyte recruitment, HSC activation via TGF-β and PDGF, collagen deposition and fibrosis, and disrupted FGF19 feedback that further amplifies BA hepatotoxicity in a self-reinforcing vicious cycle. AHR, aryl hydrocarbon receptor; BA, bile acid; CCL2/CCR2, C-C chemokine ligand 2/receptor 2; CYP7A1, Cholesterol 7α-Hydroxylase; FGF15/FGF19, fibroblast growth factor 15 (rodent)/19 (human); FXR, farnesoid X receptor; GVB, gut vascular barrier; HSC, hepatic stellate cell; IAA, indole-3-acetic acid; IL, interleukin; IPA, indole-3-propionic acid; LPS, lipopolysaccharide; MAMP, microbe-associated molecular pattern; NF-κB, nuclear factor kappa B; NLRP, NOD-like receptor pyrin domain-containing; PDGF, platelet-derived growth factor; SCFA, short-chain fatty acid; TGF-β, transforming growth factor beta; TGR5, Takeda G-protein coupled receptor 5; TLR, toll-like receptor; TNF-α, tumor necrosis factor alpha; Treg, regulatory T cell.

A recurring question is how much of the immunoactive metabolite signal is microbial versus host-derived, and whether the two are separable at all. In practice, the most informative mediators are co-produced: primary BAs are host-synthesized from cholesterol but become immunoregulatory only after microbial transformation into secondary BA (DCA, LCA, isoalloLCA), so their net effect on the Treg-Th17 axis reflects a host–microbe joint product rather than either compartment alone. SCFAs and indole derivatives are predominantly microbial in origin but act on host receptors (GPR43/GPR109A, AHR) whose expression and downstream effect are host-determined, whereas endogenous ethanol and trimethylamine illustrate microbial generation of frankly hepatotoxic species. A practical corollary is that the relevant variable is not microbial-versus-host attribution but the functional metabolite output of a given host–microbe configuration. Critically, these mediators operate within feedback amplification loops rather than as one-way inputs. Two loops are well supported: (i) an ileal-FXR→FGF15/19→hepatic-CYP7A1 loop that couples intestinal BA sensing to hepatic synthesis and, when disrupted, feeds forward on hepatic inflammation; and (ii) a barrier→translocation→hepatic-inflammation→barrier loop in which gut-derived products injure the liver, and hepatic dysfunction in turn worsens intestinal permeability and dysbiosis, establishing the self-reinforcing vicious cycle depicted in **Figure 3**. The murine specificity of part of this BA loop further underscores that the loop’s quantitative behavior is model-dependent (25–27, 33, 36, 37, 61, 75, 78, 93–100).

### 4.5 Fungal and bacterial toxins as inflammatory effectors

A newer body of work shows that specific toxins can operate as major effectors within the axis. Candidalysin, a peptide toxin secreted by *Candida albicans*, promotes alcohol-associated liver disease and is linked to epithelial type 17 responses and inflammasome activation (51–54). Cytolysin-positive *Enterococcus faecalis* has likewise been implicated in alcoholic hepatitis, and phage targeting of this pathobiont ameliorates liver disease in preclinical models. It is important to specify the level of human validation for these two exemplar toxins, because they sit at different points on the evidence spectrum. For cytolysin, the strongest human data come from a clinical cohort of patients with alcohol-associated hepatitis, in which fecal cytolysin positivity was associated with markedly increased 180-day mortality, while causal sufficiency was established in gnotobiotic mice colonized with cytolytic *E. faecalis* and reversed by precision phage therapy, together approaching the standard of a bona fide human-relevant driver, though the therapeutic arm remains preclinical (55). For candidalysin, human evidence is at present associative: patients with alcoholic hepatitis show intestinal fungal dysbiosis with Candida expansion and systemic anti-Candida immune responses, and candidalysin’s cytolytic and type-17–activating effector functions are firmly established in epithelial and murine systems, but a causal role in human disease has not yet been demonstrated in interventional studies (47, 51–54). We therefore present cytolysin as a more advanced translational candidate and candidalysin as a mechanistically defined but human-unvalidated effector, to avoid overstating the maturity of either. These observations shift the field from descriptive dysbiosis to a more precise identification of actionable microbial virulence factors and illustrate why multi-kingdom profiling, toxin-specific assays, and function-centered microbiology are more informative than broad compositional profiling alone (4, 47, 48, 55) (**Figure 3**, **Table 2**).

**Table 2:** Principal microbial products, metabolites, and host pathways that mediate gut-liver-immune communication.

Mediator/signal category	Origin	Key molecules/examples	Host receptor/sensor	Target cell(s)/tissue	Homeostatic function	Disease-promoting consequence	Key refs
MAMPs	Gut bacteria (gram-negative LPS; flagellin; bacterial DNA)	LPS, flagellin, peptidoglycan, CpG DNA	TLR4 (LPS), TLR5 (flagellin), TLR9 (CpG); NF-κB pathway	Kupffer cells, hepatocytes, LSECs, macrophages	Controlled TLR signaling maintains hepatic immune surveillance and tolerance at low portal LPS levels	Barrier failure → increased portal LPS → TLR4-driven steatohepatitis, fibrosis, HCC tumorigenesis	(8, 57, 89, 90)
Inflammasome activators	Dysbiotic bacterial products; DAMPs released during cell death	Flagellin, bacterial RNA, uric acid, ATP	NLRP3, NLRP6, AIM2 inflammasomes; caspase-1 activation; IL-18/IL-1β release	Intestinal epithelial cells, macrophages, hepatocytes	NLRP6-mediated antimicrobial secretion; IL-18 epithelial defense	Inflammasome-driven dysbiosis transmissibility in MASLD; altered microbial composition and MASLD progression	(57, 89)
Primary BAs	Hepatic synthesis from cholesterol (cholic acid (CA), chenodeoxycholic acid (CDCA))	CA, CDCA; conjugated as taurine/glycine salts (TCA, TCDCA)	FXR (nuclear), TGR5 (membrane); intestinal FXR → FGF19/FGF15 → hepatic FGFR4 feedback	Hepatocytes, cholangiocytes, intestinal epithelium, macrophages	Promotes bile flow, lipid absorption, antimicrobial defense; FXR activation suppresses *de novo* BA synthesis via FGF19	Excess primary BAs during cholestasis drive cholangiocyte injury; disrupted FXR-FGF15/19 axis promotes hepatic steatosis and inflammation	(25–27, 63)
Secondary BAs	Microbial 7α-dehydroxylation of primary BAs in the colon	Deoxycholic acid (DCA), lithocholic acid (LCA), ursodeoxycholic acid (UDCA), isoallolithocholic acid (isoalloLCA)	FXR, TGR5, VDR, RORγt (isoalloLCA → Treg induction)	Colonocytes, macrophages, Treg, NKT cells, hepatocytes	DCA/LCA restrict bacterial overgrowth; isoalloLCA promotes Treg differentiation; UDCA cytoprotection	DCA accumulation → HSC senescence → HCC promotion; excess secondary BAs in ALD → NKT accumulation via CXCL16	(25, 61, 70, 72–74)
SCFAs	Microbial fermentation of dietary fiber (primarily Firmicutes and Bacteroidetes)	Butyrate, propionate, acetate	GPR41, GPR43, GPR109A (HCAR2); HDAC inhibition (butyrate)	Colonocytes, macrophages, Tregs, hepatocytes	Butyrate: colonocyte energy source, tight-junction reinforcement, anti-inflammatory macrophage imprinting, Treg induction; propionate: hepatic lipid metabolism	SCFA depletion in dysbiosis → epithelial energy deficit, barrier weakening, Treg loss, increased intestinal and hepatic inflammation	(36, 37, 93)
Tryptophan metabolites	Microbial and host enzymatic metabolism of dietary tryptophan	Indole, indole-3-acetic acid (IAA), indole-3-propionic acid (IPA), kynurenine (IDO-mediated)	Aryl hydrocarbon receptor (AHR); pregnane X receptor (PXR)	IECs, macrophages, ILC3s, hepatocytes	Indole metabolites reinforce epithelial barrier, promote IL-22 production, AHR-mediated mucosal homeostasis	Reduced indole metabolites in ALD → diminished AHR signaling → worsened hepatic steatosis and inflammation; kynurenine pathway skew in chronic disease	(75, 78, 94, 95)
Endogenous ethanol and related metabolites	Microbial fermentation (high-alcohol-producing microbes: certain *Klebsiella pneumoniae*, *E. coli*)	Endogenous ethanol, acetaldehyde, TMAO (trimethylamine N-oxide)	Hepatic ADH/ALDH pathways; oxidative stress; TLR4 (acetaldehyde-driven)	Hepatocytes, LSECs, Kupffer cells	Low-level microbial fermentation products generally non-pathogenic under intact barrier	High-alcohol-producing *Klebsiella* linked to MASLD; TMAO linked to steatosis and cardiovascular risk; endogenous ethanol exposure replicates ALD-like hepatic injury	(96–99)
Fungal-derived toxins and PAMPs	Gut mycobiome (*Candida albicans*, *Aspergillus* spp.)	Candidalysin (peptide toxin), β-glucan, mannan	Candidalysin → EGFR/p38/epithelial IL-1α/IL-36; β-glucan → Dectin-1/CLR; mannan → TLR4/TLR2	Intestinal epithelium, neutrophils, macrophages, Th17 cells	Controlled fungal sensing shapes antimicrobial immunity; Dectin-1 signaling maintains barrier fungal containment	Candidalysin drives ALD progression via epithelial type 17 response and inflammasome activation; mycobiome dysbiosis linked to fibrosis severity and alcoholic hepatitis	(47, 48, 51–54)
Bacterial toxins and pathobiont virulence factors	Dysbiotic bacteria (*Enterococcus faecalis*, *Clostridium* spp.)	Cytolysin (*E. faecalis*), deoxycholate-linked genotoxins	Cytolysin → direct pore-forming hepatocyte death; DCA → DNA damage/senescence	Hepatocytes, biliary epithelium, colonocytes	Commensal *Enterococcus* species generally non-pathogenic; low-level colonization without liver-toxic sequelae under intact barrier	Cytolysin+ *E. faecalis* drives hepatocellular death and worsens ALD; phage targeting ameliorates liver disease in preclinical models	(55)
IgA and biliary antimicrobial programs	Liver-to-gut: hepatocyte-derived polymeric IgA; bile-contained lactoferrin and lysozyme	Secretory IgA (SIgA), biliary lactoferrin, antimicrobial BA-detergent activity	Polymeric immunoglobulin receptor (pIgR) on hepatocytes and cholangiocytes	Intestinal lumen, commensal and pathogenic bacteria	SIgA coats and immobilizes bacteria; biliary IgA limits luminal bacterial translocation; BA antimicrobial properties restrict overgrowth	Cholestasis and cirrhosis impair biliary IgA output → bacterial overgrowth → increased translocation → feed-forward hepatic injury	(3, 7, 25–27)
GVB signals	Portal endothelial cells, pericytes; regulated by commensal microbiota and BAs	PV1 (PLVAP), wnt signaling, lipid sphingosine-1-phosphate (S1P)	FXR (intestinal BA sensing → GVB integrity); Wnt/β-catenin; S1P receptors	Portal venular endothelium, portal macrophages	GVB restricts translocation of bacterial products larger than tight junctions permit; FXR activation preserves GVB integrity	GVB disruption in MASLD, ALD, and cirrhosis → bacterial product flux → LSEC and Kupffer cell activation → steatohepatitis amplification	(22, 33, 61)

ADH, alcohol dehydrogenase; AHR, aryl hydrocarbon receptor; ALD, alcohol-associated liver disease; ALDH, aldehyde dehydrogenase; BA, bile acid; CA, cholic acid; CDCA, chenodeoxycholic acid; CLR, C-type lectin receptor; DAMP, damaged-associated molecular pattern; DCA, deoxycholic acid; FGF, fibroblast growth factor; FGFR, fibroblast growth factor receptor; FXR, farnesoid X receptor; GPR, G-protein coupled receptors; GVB, gut vascular barrier; HCAR2, hydroxycarboxylic acid receptor 2; HCC, hepatocellular carcinoma; HDAC, histone deacetylase; HSC, hepatic stellate cell; IAA, indole-3-acetic acid; IDO, indoleamine 2,3-dioxygenase; IEC, intestinal epithelial cell; IgA, immunoglobulin A; IL, interleukin; ILC3, group 3 innate lymphoid cell; IPA, indole-3-propionic acid; isoalloLCA, isoallolithocholic acid; LCA, lithocholic acid; LPS, lipopolysaccharide; LSEC, liver sinusoidal endothelial cell; MAMP, microbe-associated molecular pattern; MASLD, metabolic dysfunction-associated steatotic liver disease; NF-κB, nuclear factor kappa B; NKT, natural killer T cell; NLRP, NOD-like receptor pyrin domain; PAMP, pathogen-associated molecular pattern; pIgR, polymeric immunoglobulin receptor; PLVAP, plasmalemma vesicle-associated protein; PV1, pleiotrophin variant 1; PXR, pregnane X receptor; RORγt, RAR-related orphan receptor gamma t; SCFA, short-chain fatty acid; SIgA, secretory Immunoglobulin A; S1P, sphingosine-1-phosphate; TCA, taurocholic acid; TCDCA, taurochenodeoxycholic acid; TGR5, Takeda G-protein receptor 5; TLR, toll-like receptor; TMAO, trimethylamine N-oxide; Treg, regulatory T cell; UDCA, ursodeoxycholic acid; VDR, vitamin D receptor.

## 5 Disease-specific manifestations of the gut-liver-immune axis

Although the following sections are organized by diagnosis, several core mechanisms recur across diseases but contribute differently depending on stage, and it is useful to resolve them along an early-inflammatory → fibrotic → advanced/oncogenic trajectory. Inflammasome activation (NLRP3/NLRP6) is most decisive early, where it shapes microbial composition and initiates IL-1β/IL-18–driven steatohepatitis; its later contribution is increasingly indirect, operating through sustained myeloid activation. GVB disruption is similarly an upstream, initiating event, experimentally shown to precede steatohepatitis, but in advanced cirrhosis it becomes part of a severe, self-amplifying translocation state with systemic endotoxemia rather than a discrete trigger. NET formation is comparatively stage-restricted, becoming prominent in acute inflammatory exacerbations such as alcoholic hepatitis and in decompensated cirrhosis, where neutrophil-derived traps amplify hepatic injury and contribute to organ failure, while playing little role in quiescent steatosis. SCFA and indole signaling, by contrast, are protective pathways whose loss is most consequential early (permitting barrier weakening and Treg deficit) and whose restoration is most plausible before irreversible architectural remodeling; in established fibrosis or cirrhosis, supplying these metabolites is less likely to reverse fixed structural disease. This stage-resolved view (summarized across **Tables 2**, **3**) clarifies why a mechanism can be a rational therapeutic target at one stage and an ineffective or even harmful one at another (10, 13, 22, 36, 37, 57, 62, 78, 85, 93–95).

**Table 3:** Disease-specific immune signatures and dominant gut-liver-immune mechanisms across major liver disorders.

Disease	Key microbial/ecological alterations	Barrier dysfunction	Dominant molecular mediators	Primary immune-cell effectors	Main downstream hepatic consequence	Evidence level	Key refs
MASLD/MASH	↓ Firmicutes/Bacteroidetes ratio; ↑ Proteobacteria; altered mycobiome; high-alcohol-producing microbes (*Klebsiella*); virome changes linked to fibrosis severity	Tight-junction disruption; GVB disruption; ↑ intestinal permeability (biomarkers: zonulin, LBP); GVB disruption precedes steatohepatitis in animal models	LPS (TLR4), inflammasome activation (NLRP3/6), endogenous ethanol, altered primary/secondary BA pools, ↓ SCFAs, ↓ indole metabolites	Kupffer cells and recruited macrophages; MAIT cells (activated by gut-derived riboflavin metabolites); Th17 ↑/Treg ↓ balance; NKT cells	Hepatic steatosis → steatohepatitis → fibrosis; impaired immune tolerance; HCC predisposition	Strong (mechanistic in mouse models; associative in human metagenomics, metabolomics)	(8, 17, 22, 45, 46, 55, 57, 69, 76, 85, 93–99, 101, 102)
ALD/AH	↓ Bacteroidetes; ↑ Proteobacteria; cytolysin+ *E. faecalis* enrichment; gut mycobiome expansion (*Candida*); altered virome; SIBO	Ethanol directly disrupts tight junctions (occludin, claudin-1); mucus layer thinning; GVB failure; ↑↑ portal LPS	LPS (TLR4), candidalysin (IL-17/EGFR), cytolysin (*E. faecalis*), ↓ FGF19, altered BA pool, ↓ tryptophan/indole metabolites	Neutrophils (paradoxical hyperactivation + dysfunction); Kupffer cells; MAIT cells (depleted or dysfunctional); Th17 ↑; NK cells (impaired)	Acute alcoholic hepatitis; steatohepatitis; fibrosis/cirrhosis; infection susceptibility; systemic immune dysfunction	Strong (human clinical, mechanistic animal models, cytolysin phage therapy)	(10, 13, 47, 48, 51–55, 58, 63, 64, 92, 106–111)
PSC+IBD-Associated Hepatobiliary Disease	Altered intestinal microbiota; reduced microbial diversity; ↑ *Fusobacterium*; gut-primed immune cell imprinting even with quiescent IBD	IBD-associated epithelial barrier disruption; aberrant gut-homing endothelial programs in hepatic portal tracts (CCL25 upregulation)	Aberrant CCL25/CCR9 and integrin-mediated gut-lymphocyte hepatic homing; altered BA composition (cholestatic BAs); endothelial activation	Gut-primed CCR9^+^ lymphocytes homing to liver; Th17 cells at portal tracts; activated CD8^+^ T cells; NK cells (reduced antifibrotic function)	Biliary stricturing; secondary biliary cirrhosis; cholangiocarcinoma predisposition	Strong for trafficking (human immunology); moderate for causal microbiota role	(11, 12, 14, 79–82)
Cirrhosis and AD	SIBO; ↑ Proteobacteria; ↑ oral-origin bacteria in gut; altered mycobiome; reduced microbial diversity; microbiota composition predicts hepatic decompensation and mortality	Severe GVB and epithelial barrier dysfunction; ↑↑ endotoxemia; SIBO → ↑ translocation of intact bacteria; biliary IgA impairment	LPS, flagellin, bacterial DNA (systemic translocation); ↓↓ secondary BAs; altered FXR-FGF19 axis; ↓ intestinal motility	Neutrophils (functional exhaustion + paradoxical activation); NK cells (impaired); MAIT cells (depleted from blood, dysregulated in tissues); macrophages (monocytic shift)	HE; SBP and systemic infections; acute-on-chronic liver failure (ACLF); extrahepatic organ dysfunction	Strong (human clinical cohorts, multi-omic prognostic models)	(13, 33, 49, 61, 62, 64, 87, 88, 112, 114–121, 144–146)
HCC	HCC-associated gut microbiota signatures in MASLD-cirrhosis; ↑ *Clostridium*; altered secondary BA metabolism; *E. faecalis* translocation; microbiota shapes immunotherapy response	Chronic barrier dysfunction → sustained carcinogenic microbial product flux; DCA-driven colonocyte senescence and portal delivery	TLR4-driven hepatocyte proliferative signaling; DCA → HSC senescence SASP; microbiota-CXCL16-NKT axis; LPS → tumor-promoting inflammation	NKT cells (accumulation driven by microbiota-BA-CXCL16 circuit → tumor promotion OR immune surveillance, context-dependent); NK cells; tumor-associated macrophages; CD8^+^ T cells (exhaustion in immunosuppressive niche)	HCC development in cirrhotic liver; tumor immune evasion; differential immunotherapy response by microbiota status	Moderate–Strong (mechanistic in mouse; associative in human cohorts)	(14, 70, 71, 89, 90, 119–124)

ACLF, acute-on-chronic liver failure; AD, acute decompensation; ALD, alcohol-associated liver disease; AH, alcoholic hepatitis; BA, bile acid; CCL, C-C chemokine ligand; CCR, C-C chemokine receptor; CD, cluster of differentiation; CXCL, C-X-C chemokine ligand; DCA, deoxycholic acid; EGFR, epidermal growth factor receptor; FGF19, fibroblast growth factor 19; FXR, farnesoid X receptor; GVB, gut vascular barrier; HCC, hepatocellular carcinoma; HE, hepatic encephalopathy; HSC, hepatic stellate cell; IBD, inflammatory bowel disease; IgA, immunoglobulin A; IL, interleukin; LBP, LPS-binding protein; LPS, lipopolysaccharide; MAIT, mucosal-associated invariant T cell; MASH, metabolic dysfunction-associated steatohepatitis; MASLD, metabolic dysfunction-associated steatotic liver disease; NK, natural killer; NKT, natural killer T; NLRP, NOD-like receptor pyrin domain; PSC, primary sclerosing cholangitis; SASP, senescence-associated secretory phenotype; SBP, spontaneous bacterial peritonitis; SCFA, short-chain fatty acid; SIBO, small intestinal bacterial overgrowth; TLR, toll-like receptor; Treg, regulatory T cell.

### 5.1 MASLD and MASH

MASLD/MASH is one of the clearest examples of how metabolic stress, barrier dysfunction, microbial products, and immune activation converge. High-fat or Western-type diets can alter microbial composition, increase intestinal permeability, change BA metabolism, and enhance the delivery of inflammatory mediators to the liver. Clinical and metagenomic studies have shown associations between advanced fibrosis and gut microbial signatures, altered mycobiome and virome composition, endogenous ethanol pathways, and abnormal BA profiles (45, 46, 76, 96, 101–103).

Mechanistically, MASLD/MASH is not simply fat accumulation plus inflammation. Rather, excess nutrient load intersects with microbiota-derived signals that modify macrophage activation, T-cell polarization, and stellate-cell activity. Experimental evidence supports a role for inflammasome-dependent dysbiosis, GVB disruption, tight-junction loss, altered endogenous ethanol production, and FXR-related pathways in promoting steatohepatitis and fibrosis (22, 57, 85, 97, 98, 104, 105). The multiplicity of these pathways helps explain the heterogeneity of MASLD and why no single microbial biomarker has yet proven sufficient for clinical stratification.

Immune cells are central to this transition from steatosis to steatohepatitis. Macrophages bridge microbial sensing to cytokine production and fibrogenesis, while adaptive immune shifts and MAIT cell activation may contribute to persistent inflammatory tone and barrier dysfunction (8, 9, 17, 69). Protective metabolites such as indole and short-chain fatty acids point in the opposite direction and support the notion that restoring immune education rather than merely suppressing inflammation may be a rational strategy in metabolically driven disease (93–95).

### 5.2 ALD

ALD is a prototypic gut-liver-immune disorder because ethanol directly injures the intestinal barrier, changes microbiota composition, alters BA signaling, and amplifies portal exposure to microbial products (10, 13, 58, 106, 107). In severe disease, fungal dysbiosis, cytolysin-positive bacteria, candidalysin, and virome changes further intensify the inflammatory burden (47, 48, 51, 55, 108).

The immune consequences of these changes are substantial. Kupffer cells and infiltrating myeloid cells are activated by microbial products and produce cytokines, chemokines, and reactive intermediates that drive tissue damage. Neutrophils become abundant, yet host defense is often defective, especially in advanced disease. MAIT cell dysfunction in alcoholic liver disease is a particularly important illustration of how leaky gut, microbial contact, and altered hepatic immunity can combine to impair antibacterial responses while sustaining inflammation (14, 64, 66).

Alcoholic hepatitis is associated with disturbed BA and fibroblast growth factor (FGF)19 profiles, while preclinical studies show that modulating the intestinal BA-FXR-FGF15 axis can improve alcohol-induced liver disease (63, 92). Other therapeutic strategies, including tributyrin supplementation, IL-22-producing engineered bacteria, restoration of *Akkermansia muciniphila*, and phage therapy directed at pathobionts, further support the model that ALD progression is tightly linked to the gut-liver-immune axis rather than being driven by ethanol toxicity alone (55, 109–111).

### 5.3 PSC and IBD-associated hepatobiliary disease

PSC is especially informative because it highlights inter-organ immune trafficking as a disease mechanism. The close epidemiological relationship between PSC and IBD, the predominance of cholangiopathy, and the frequent mismatch between intestinal and hepatic disease activity have long suggested that immune communication between gut and liver is central to pathogenesis (11, 12). Experimental and human studies support a model in which intestinal inflammation, microbial perturbation, and altered trafficking cues promote the recruitment of gut-imprinted lymphocytes to the liver. The demonstration that hepatic endothelial CCL25 can recruit CCR9+ gut-homing lymphocytes to the liver remains a landmark observation in this field (79, 80).

Subsequent work has expanded that model to a more complex trafficking and imprinting framework involving integrins, chemokines, endothelial activation, and non-endstage alterations in gut-homing programs (14, 81, 82). Microbial and BA abnormalities are also relevant to PSC, although their precise relationships to immune trafficking and cholangiocyte injury remain incompletely resolved. Nonetheless, PSC illustrates better than perhaps any other liver disease that the gut-liver-immune axis cannot be explained solely by soluble mediators; migratory immune cells can themselves be disease vectors.

This perspective has therapeutic implications. If PSC partly reflects misdirected mucosal immunity, then therapies targeting trafficking, endothelial cues, or intestinal immune imprinting may be more rational than broad immunosuppression alone. The same logic may also apply to selected cases of immune checkpoint hepatitis, autoimmunity, and post-transplant immune complications, although the evidence base is much stronger in PSC than in other settings (12, 14, 81). Because PSC is the clearest prototype of misdirected mucosal immunity, gut-educated lymphocytes producing disease at a hepatic site, it is also the most rational setting in which to propose biomarkers of this process. A candidate panel would combine (i) circulating frequencies and activation states of gut-imprinted lymphocytes bearing CCR9 and α4β7, whose aberrant hepatic homing is mechanistically central; (ii) MAIT-cell phenotype, given the consistent blood-versus-tissue redistribution observed across chronic liver disease; (iii) soluble or endothelial expression of the cognate homing ligands MAdCAM-1 and CCL25, which mark the trafficking axis itself; and (iv) gut-homing transcriptional imprinting signatures detectable in peripheral or hepatic lymphocytes. Such markers would aim to capture the direction of mucosal immune output rather than its mere magnitude, and if validated longitudinally against tissue could in principle identify patients in whom trafficking-directed therapy (anti-α4β7, anti-CCR9) is most rational and stratify trafficking activity before irreversible biliary damage. We emphasize that this panel remains a hypothesis requiring prospective validation, and that blood-based trafficking markers must be interpreted against the blood-tissue discordance (12, 14, 79–82).

### 5.4 Cirrhosis and AD

Cirrhosis represents a late-stage convergence of multiple gut-liver-immune abnormalities. Patients commonly exhibit small intestinal bacterial overgrowth, endotoxemia, fungal dysbiosis, altered BA metabolism, and profound immune dysfunction (49, 61, 64, 87, 88). The microbiota in cirrhosis has been linked to hepatic encephalopathy (HE), decompensation, and extrahepatic complications, with both bacterial and viral signals participating in disease progression (13, 112). Although these complications co-occur, the gut-derived factors that drive them are mechanistically dissociable and separating them clarifies why a single microbiota-directed intervention may affect one complication more than another. Hepatic encephalopathy is driven principally by gut-generated neuroactive and nitrogenous products, above all ammonia, produced by bacterial urease activity and intestinal glutaminase and inadequately detoxified by the failing liver and shunted circulation, together with other microbially modulated mediators; this is why ammonia-lowering, microbiota-directed therapies such as lactulose and rifaximin are effective in HE specifically (113). The systemic inflammatory milieu, by contrast, is driven chiefly by translocation of intact PAMPs (LPS, bacterial DNA, flagellin) that reach the systemic circulation through a severely compromised gut barrier and sustain a chronic, low-grade activation of circulating and hepatic immune cells. Infection risk arises from the conjunction of this increased translocation with cirrhosis-associated immune dysfunction: the same barrier failure that delivers microbial products coincides with impaired neutrophil and monocyte effector function and reduced antimicrobial clearance, so that exposure is increased precisely when defense is weakest (13, 62, 64, 87, 88). Thus, HE reflects predominantly a metabolite/neurotoxin problem, systemic inflammation a PAMP-translocation problem, and infection a translocation plus immune paralysis problem, distinctions with direct therapeutic implications. Importantly, cirrhosis-associated immune dysfunction combines exaggerated inflammatory signaling with impaired antimicrobial responses, thereby increasing vulnerability to infection and organ failure.

Experimental data show that FXR regulates entry sites for bacterial translocation through the GVB, while clinical studies support roles for altered BAs, dysmotility, and microbial overgrowth in facilitating systemic exposure to intestinal products (33, 61, 62). This framework helps explain why decompensated cirrhosis behaves as a systemic inflammatory disease as much as a localized liver disorder.

The therapeutic implications are substantial. In cirrhosis, microbiota-directed interventions have shown promise for selected endpoints, but safety, engraftment variability, and host immune context remain major determinants of response (114–118). Thus, even when the microbiome is targeted directly, the outcome is inseparable from the immune and barrier state of the host.

### 5.5 HCC

HCC emerges within a chronically remodeled immune and metabolic niche, and the gut-liver-immune axis contributes to this niche at multiple levels. Human studies have identified gut microbiota signatures associated with HCC and with HCC arising in MASLD or cirrhosis, supporting the translational relevance of these mechanisms (71, 119–121).

Mechanistic evidence is strongest for pathways involving endotoxin/TLR4 signaling, microbial metabolites, Enterococcus pathobionts, and BA-dependent control of NKT-cell surveillance. Obesity-associated microbial metabolites such as deoxycholic acid (DCA) can promote carcinogenesis through senescence-associated secretory phenotype (SASP), while gut microbiota-mediated BA metabolism influences hepatic chemokine signals and NKT-cell accumulation that shape tumor control (70, 89, 90, 122). Additional work implicates chronic liver disease-enabled colonization by *Enterococcus faecalis* and pathobiont translocation as tumor-promoting events (123, 124).

**Figure 4:**
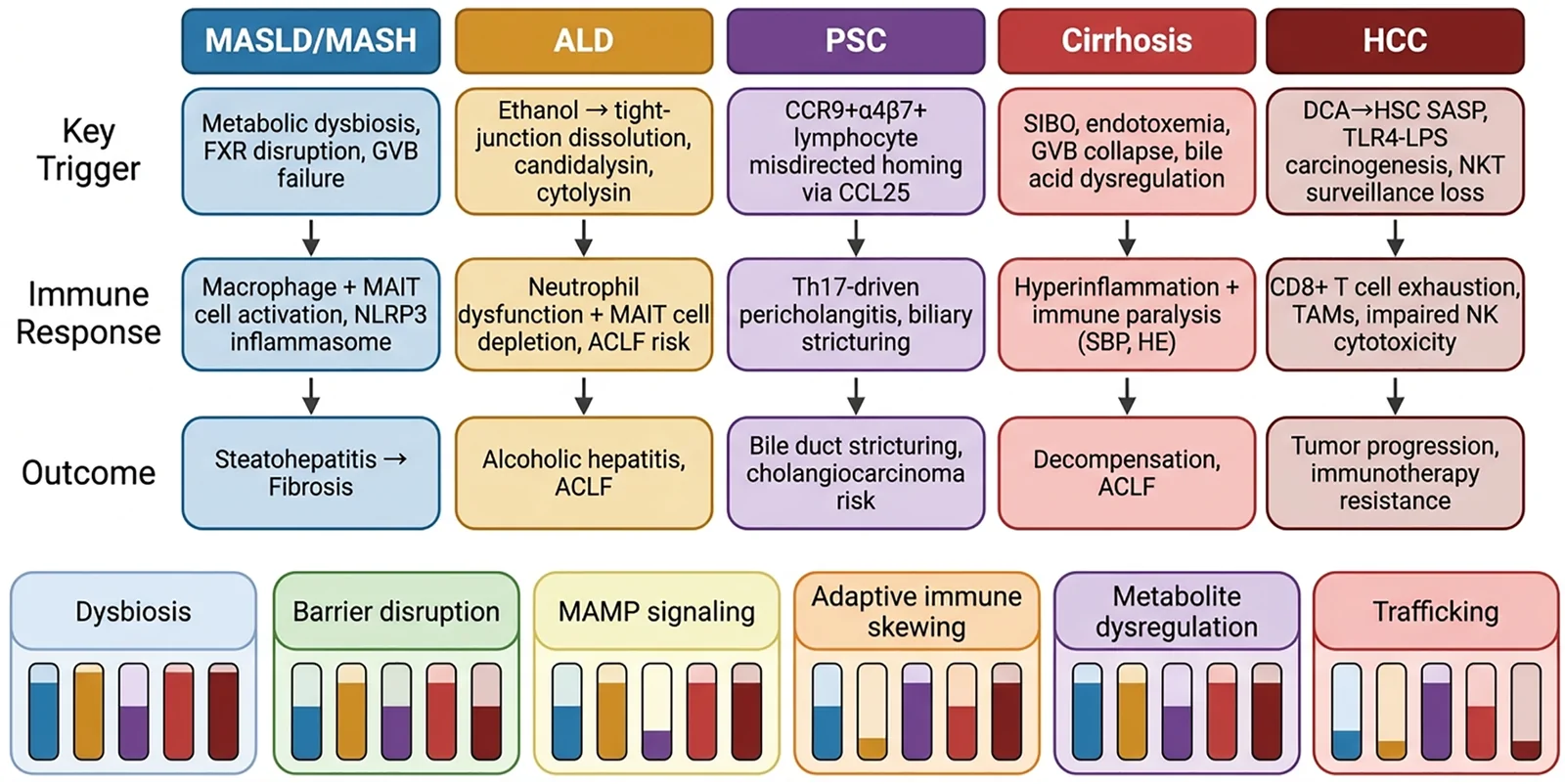
Disease-specific manifestations of gut-liver-immune dysregulation across five major hepatic disorders. Five parallel disease columns present dominant ecological trigger (row 1), immune response (row 2), and tissue outcome (row 3) for each disorder. MASLD/MASH: metabolic dysbiosis with FXR-FGF19 axis disruption and GVB failure leads to portal LPS accumulation, NLRP3 inflammasome activation, and macrophage and MAIT cell-mediated steatohepatitis progressing to fibrosis. ALD: ethanol-driven tight-junction dissolution, candidalysin from *Candida*, and cytolysin from *E. faecalis* amplify neutrophilic and macrophage-mediated hepatocellular injury; MAIT-cell depletion from blood and dual neutrophil dysfunction phenotype contribute to ACLF risk. PSC: CCR9^+^α4β7^+^ lymphocytes aberrantly home to hepatic portal tracts via CCL25-mediated endothelial signaling, driving Th17-associated pericholangitis and biliary stricturing independent of intestinal IBD activity. Cirrhosis: convergence of SIBO, severe GVB failure, depleted biliary SIgA, and altered BA antimicrobial output produces the immune paradox of concurrent hyperinflammation and immune paralysis, elevating risk of SBP, HE, and ACLF. HCC: microbiota-derived DCA induces SASP in HSCs, CXCL16-mediated NKT accumulation disrupts tumor surveillance, and LPS-TLR4 signaling promotes hepatocyte proliferation in a tumor microenvironment characterized by CD8^+^ T-cell exhaustion, immunosuppressive TAMs, and impaired NK cytotoxicity. A bottom comparison strip rates the relative contribution of six pathological modules (dysbiosis, barrier disruption, MAMP signaling, adaptive immune skewing, metabolite dysregulation, and immune-cell trafficking) across all five diseases. ACLF, acute-on-chronic liver failure; ALD, alcohol-associated liver disease; BA, bile acid; CCL, C-C chemokine ligand; CCR, C-C chemokine receptor; CD, cluster of differentiation; CXCL, C-X-C chemokine ligand; DCA, deoxycholic acid; FGF19, fibroblast growth factor 19 (human); FXR, farnesoid X receptor; GVB, gut vascular barrier; HCC, hepatocellular carcinoma; HE, hepatic encephalopathy; HSC, hepatic stellate cell; IBD, inflammatory bowel disease; LPS, lipopolysaccharide; MAIT, mucosal-associated invariant T cell; MAMP, microbe-associated molecular pattern; MASH, metabolic dysfunction-associated steatohepatitis; MASLD, metabolic dysfunction-associated steatotic liver disease; NK, natural killer; NKT, natural killer T cell; NLRP, NOD-like receptor pyrin domain-containing; PSC, primary sclerosing cholangitis; SASP, senescence-associated secretory phenotype; SBP, spontaneous bacterial peritonitis; SIBO, small intestinal bacterial overgrowth; SIgA, secretory immunoglobulin A; TAM, tumor-associated macrophage; TLR4, toll-like receptor 4.

A question of increasing importance is whether microbiota-associated mechanisms can be assigned to distinct phases of hepatocarcinogenesis, initiation, promotion, and progression, rather than treated as a single tumor-promoting influence. At the initiation phase, gut-derived signals contribute genotoxic and pro-survival inputs: the secondary bile acid DCA and other microbial products can promote DNA damage, while endotoxin accumulation and TLR4 signaling have been shown to prevent carcinogen-induced apoptosis and favor survival of initiated hepatocytes (89, 90, 122). At the promotion phase, the dominant mechanisms are inflammatory and senescence-associated rather than directly mutagenic: DCA-induced SASP in hepatic stellate cells, sustained TLR4/NF-κB inflammatory signaling, and subversion of immune surveillance through the microbiota-BA-CXCL16-NKT axis collectively expand a pro-tumor niche (70, 89, 122). At the progression and immune-evasion phase, microbiota- and BA-shaped remodeling of the tumor microenvironment, immunosuppressive tumor-associated macrophages, exhausted CD8+ T cells, and impaired NK cytotoxicity, governs tumor growth and may influence immunotherapy responsiveness (14, 70, 71, 123, 124). This phase-resolved framework is necessarily provisional, because much of the initiation- and promotion-phase evidence is causal in rodent models but remains associative in humans (125) nonetheless, distinguishing the phases is valuable because interventions plausibly effective in prevention (barrier/microbiota/BA modulation, acting on initiation and promotion) differ from those relevant to established tumors (immune-directed therapy, acting on progression). These studies show that microbiota-associated carcinogenesis is mediated through immune and stromal remodeling rather than direct hepatocyte toxicity alone, and that microbiota composition, BA state, and immune-cell distribution may influence responses to immunotherapy in established HCC (14, 70, 71) (**Figure 4**, **Table 3**).

## 6 Therapeutic opportunities and future directions

### 6.1 Microbiota-directed and barrier-directed interventions

Before discussing individual nodes, it is useful to state explicitly which mediators are presently actionable in the clinic versus predominantly experimental, because the gut-liver-immune literature often conflates mechanistic plausibility with clinical readiness. Clinically actionable interventions, those with regulatory approval or controlled human trial data for a liver-relevant endpoint, currently include the thyroid hormone receptor-β agonist resmetirom (approved for MASH with fibrosis), FXR agonists such as obeticholic acid (phase 3 fibrosis data, with tolerability constraints), ursodeoxycholic acid (established in cholestatic disease), rifaximin (selective intestinal decontamination in hepatic encephalopathy), and fecal microbiota transplantation in selected alcohol use disorder (AUD)/cirrhosis settings. Predominantly experimental strategies, mechanistically compelling but lacking human interventional validation, include bacteriophage therapy against cytolysin-positive *E. faecalis*, engineered live biotherapeutics (e.g., IL-22–producing bacteria), anti-candidalysin neutralization, and immunomodulatory bile acid metabolites such as isoalloLCA. The sections below and **Table 4** retain this distinction by labeling the evidence level (preclinical, phase 2, phase 3/approved) for each strategy, so that mechanistic attractiveness is not mistaken for translational maturity (55, 63, 92, 109–111, 114–118, 126–130).

**Table 4:** Therapeutic strategies targeting the gut-liver-immune axis and key translational considerations.

Therapeutic category	Strategy/agent	Target node of the axis	Mechanism of action	Disease context/evidence	Key limitations and translational considerations	Key refs
Microbiota-Directed Therapies	Fecal Microbiota Transplantation (FMT)	Microbial ecology; dysbiosis correction	Transfer of donor microbial community to restructure recipient dysbiotic microbiome; restore protective commensals, displace pathobionts	ALD (AUD): RCT evidence for clinical improvement; cirrhosis: modified microbial composition, selected endpoint benefit; HE: emerging evidence	Variable inter-individual engraftment; MDR organism transmission risk; safety concerns in advanced cirrhosis; efficacy depends on disease stage and host immune context; requires identification of optimal donor	(114–118, 131, 132)
	Phage therapy (targeted pathobiont elimination)	Specific pathobiont (cytolysin+ *E. faecalis*)	Bacteriophage cocktail targeting cytolysin+ *E. faecalis*; reduces hepatotoxic cytolysin load without broad microbiome disruption	ALD (preclinical): phage therapy ameliorated liver disease in murine ALD model; human trials pending	Currently preclinical; narrow host range requires pathobiont identification; phage resistance emergence; no human data yet	(55)
	Engineered bacteria/live biotherapeutics	Gut microbial ecology; specific metabolite production	Genetically engineered bacteria programmed to produce IL-22, butyrate, or other protective molecules *in situ*; restore specific microbial functions	ALD, MASLD (preclinical): engineered IL-22-producing bacteria and *Akkermansia muciniphila* restoration improve liver disease	Safety and regulatory pathway for GMO-based live therapeutics; engraftment and persistence uncertain in disease context; host immune acceptance unclear	(109–111, 126–128)
Barrier-Directed Therapies	Butyrate/tributyrin supplementation	Epithelial barrier; macrophage phenotype; Treg induction	SCFA supplementation restores colonocyte energy, tight-junction proteins, anti-inflammatory macrophage imprinting, and Treg differentiation	ALD (preclinical): tributyrin improves gut barrier and liver pathology; MASLD (associative in humans); no large RCTs yet	Bioavailability of oral SCFAs limited (rapid colonocyte absorption); tributyrin as prodrug improves delivery but lacks clinical trial data; barrier phenotyping needed to identify responders	(36, 93, 107, 109)
	GVB restoration	Portal endothelium; GVB integrity	FXR agonist-mediated GVB stabilization; microbiota-mediated Wnt signaling support; barrier-targeted drug delivery	MASLD, ALD, cirrhosis (preclinical): FXR activation prevents GVB disruption and reduces bacterial translocation	Systemic FXR agonist side effects (dyslipidemia with OCA); GVB is heterogeneous, barrier phenotyping must guide patient selection; human validation needed	(22, 33, 61)
	Mucus layer and antimicrobial restoration (*Akkermansia muciniphila*)	Mucus barrier; epithelial interface	*A. muciniphila* restores mucus layer thickness, tight-junction integrity, and mucosal immune homeostasis; can be administered as pasteurized preparation	MASLD, obesity, ALD (clinical and preclinical): pasteurized *A. muciniphila* shows metabolic benefit and improved barrier function in human trials	Mechanism is partially independent of live bacteria (pasteurized form sufficient); long-term engraftment data limited; efficacy in advanced fibrosis unknown	(110, 111)
BA–Directed Therapies	FXR agonists (OCA, cilofexor, tropifexor)	BA signaling hub; hepatic and intestinal FXR	Activate hepatic and intestinal FXR → ↓ BA synthesis (via FGF19), ↑ BA transport, anti-inflammatory and antifibrotic hepatic signaling, GVB stabilization	MASH: Phase II/III trials (OCA improves fibrosis); ALD (FXR-FGF15 axis modulation improves preclinical ALD); PSC (trials ongoing)	OCA: pruritus, dose-dependent LDL elevation; intestinal FXR vs. hepatic FXR target selectivity matters; patient stratification by BA pool composition recommended	(25, 63, 92, 104, 105)
	TGR5 agonists	Macrophage and epithelial TGR5 signaling	Activate TGR5 on macrophages → ↓ NF-κB inflammatory signaling; TGR5 on enteroendocrine cells → GLP-1 release → improved metabolic signaling	MASLD/metabolic disease (preclinical); combined FXR/TGR5 agonism explored in MASH trials	Limited selective TGR5 agonists in advanced clinical trials; gallbladder side effects (motor relaxation); systemic vs. intestinal selectivity needed	(25, 27)
	Secondary BA metabolite supplementation (isoalloLCA, UDCA)	Treg differentiation; BA immunomodulation	isoalloLCA promotes FoxP3^+^ Treg differentiation via mitochondrial ROS pathway; UDCA cytoprotection and anti-apoptotic effects in cholangiocytes	PSC (UDCA: clinical use, anti-cholestatic); Treg axis modulation (preclinical isoalloLCA data)	High-dose UDCA potentially harmful in PSC; isoalloLCA human data limited; optimal BA species, doses, and patient phenotypes require clinical validation	(72–74, 91)
Metabolite-Directed Therapies	Tryptophan/indole supplementation or AHR pathway activation	AHR-mediated mucosal homeostasis; epithelial IL-22 production; barrier repair	IAA, IPA, and other AHR ligands reinforce epithelial tight junctions, promote ILC3/Th22-mediated IL-22 production, reduce hepatic steatosis and inflammation	ALD, MASLD (preclinical): indole metabolite supplementation reduces hepatic steatosis and barrier disruption	Intestinal microbiota variability determines endogenous indole production; systemic AHR activation has immunosuppressive risks; bioavailability and delivery route need optimization	(75, 78, 94, 95)
	SCFA-producing dietary interventions (high-fiber diet, prebiotics)	Microbial ecology; SCFA production; Treg induction	Dietary fiber selectively feeds SCFA-producing commensals (*Bifidobacterium*, *Faecalibacterium prausnitzii*); increases colonic butyrate and propionate; supports Treg axis and barrier integrity	MASLD (human dietary intervention studies, associative); ALD (preclinical); broader liver disease (ongoing trials)	Dietary compliance challenging; individual microbiome composition determines SCFA yield from fiber; effect size in advanced disease likely modest without additional intervention	(36, 37, 93)
Immune-Directed Therapies	Macrophage reprogramming (CSF1R inhibitors, IL-34 blockade, CCR2/CCR5 antagonism: cenicriviroc)	Hepatic macrophage recruitment and activation	CCR2/CCR5 blockade reduces monocyte-derived macrophage recruitment to liver; CSF1R inhibition depletes profibrotic macrophage pools; reprograms hepatic immune environment	MASH (cenicriviroc Phase II: anti-inflammatory benefit without clear antifibrotic benefit at 1 year); ongoing trials with combination approaches	Broad macrophage targeting risks impaired host defense (bacterial and fungal infections); anti-inflammatory vs. antifibrotic timing matters; requires companion diagnostics for macrophage phenotype	(8, 9, 14)
	Lymphocyte trafficking inhibition (anti-integrin, anti-CCR9)	Gut-to-liver lymphocyte trafficking (PSC/IBD-liver axis)	Block α4β7 integrin (vedolizumab) or CCR9 (Vercirnon/CCX282-B) to prevent gut-imprinted lymphocytes from aberrantly homing to hepatic portal tracts	PSC+IBD (ongoing trials with vedolizumab; early clinical data suggest some benefit in IBD-associated hepatic inflammation); PSC-specific CCR9 targeting being investigated	Partial efficacy in IBD (vedolizumab) may not translate to full PSC benefit; trafficking inhibition may be most effective before irreversible biliary damage; biomarker of trafficking activity needed to select patients	(11, 12, 14, 79–82)
Precision Endotyping/Multi-Omic Patient Stratification	Integrated biomarker panels (metagenomics + metabolomics + immune phenotyping + barrier markers)	Upstream identification of dominant pathological node (dysbiosis, barrier, BA, trafficking, or immune)	Combining gut microbiome composition, stool/serum metabolomics, BA profiling, barrier markers (LBP, FABP2, zonulin), and circulating immune-cell phenotyping into endotype-defining clusters	MASLD, ALD, cirrhosis (multi-omic prognostic models validated in human cohorts for decompensation and mortality risk)	No validated clinical-grade multi-omic endotyping panel yet; longitudinal sampling required; cost and complexity limit routine use; requires prospective validation in therapeutic trial cohorts	(2–6, 8, 15, 144–146)

AHR, aryl hydrocarbon receptor; ALD, alcohol-associated liver disease; AUD, alcohol use disorder; BA, bile acid; CCR, C-C chemokine receptor; CSF1R, colony stimulating factor 1 receptor; FABP2, fatty acid binding protein 2; FGF, fibroblast growth factor; FMT, fecal microbiota transplantation; FoxP3, Forkhead box P3; FXR, farnesoid X receptor; GLP-1, glucagon-like peptide-1; GMO, genetically modified organism; GVB, gut vascular barrier; HE, hepatic encephalopathy; IAA, Indole-3-acetic acid; IBD, inflammatory bowel disease; IL, interleukin; ILC3, group 3 innate lymphoid cell; IPA, indole-3-propionic acid; isoalloLCA, isoallolithocholic acid; LBP, LPS-binding protein; LDL, low-density lipoprotein; MASH, metabolic dysfunction-associated steatohepatitis; MASLD, metabolic dysfunction-associated steatotic liver disease; MDR, multidrug resistant; NF-κB, nuclear factor kappa B; OCA, obeticholic acid; PSC, primary sclerosing cholangitis; RCT, randomized controlled trial; ROS, reactive oxygen species; SCFA, short-chain fatty acid; TGR5, Takeda G-protein receptor 5; Treg, regulatory T cell; UDCA, ursodeoxycholic acid.

Microbiota-directed therapy has progressed from (70) broad probiotic concepts to more targeted approaches involving fecal microbiota transplantation, engineered bacteria, phages, and metabolite replacement. Clinical studies in AUD and cirrhosis suggest that fecal microbiota transplantation (FMT) can modify microbial composition and selected clinical endpoints, but inter-individual engraftment varies substantially and safety concerns remain, including transmission of multidrug-resistant (MDR) organisms (114–118, 131, 132). These limitations reinforce the need to define which disease endotypes are most likely to respond to microbial transfer.

Barrier-directed approaches are conceptually attractive because they target an upstream defect shared across several liver diseases. Strategies such as restoring antimicrobial peptides, butyrate availability, mucus support, or GVB integrity have shown promise in experimental systems (22, 33, 107, 109). In practice, however, barrier dysfunction is heterogeneous and may not dominate every patient’s disease trajectory. For this reason, future translational work should link barrier phenotyping to immune and metabolite profiling rather than treating intestinal permeability as a single universal defect.

### 6.2 Targeting BAs, metabolites, and phage-sensitive pathobionts

Because BAs sit at the intersection of microbial ecology, epithelial defense, metabolism, and immunity, they represent one of the most promising therapeutic nodes. FXR-directed interventions, strategies that normalize BA pools, and approaches that enhance beneficial BA metabolites may influence both local gut immunity and hepatic inflammation (25, 63, 72, 92, 104, 105). Supplementation or restoration of beneficial microbial metabolites such as indole, butyrate, or tryptophan-derived ligands could theoretically promote tolerance and barrier repair without relying on global immunosuppression (78, 93–95).

The clinical maturity of these nodes is now established. In the phase 3 regenerate trial, the FXR agonist obeticholic acid achieved fibrosis improvement without MASH worsening, albeit with dose-dependent pruritus and LDL elevation that have constrained its regulatory path (129). More decisively, the thyroid hormone receptor-β agonist resmetirom became, in the phase 3 MAESTRO-MASH trial, the first agent to meet both MASH-resolution and fibrosis-improvement endpoints and to receive regulatory approval for MASH with fibrosis, validating that pharmacologic correction of a metabolic-hepatic node can yield histological benefit (130). These trials are mechanistically relevant to the gut-liver-immune axis because FXR signaling simultaneously governs hepatic synthesis, intestinal antimicrobial defense, and GVB integrity, linking a metabolic target to barrier and immune outcomes.

More precise targeting of pathobionts is also moving from concept to experiment. Phage therapy directed at *Enterococcus faecalis* improved experimental alcoholic liver disease, and engineered bacterial platforms raise the possibility that future therapy might reprogram the gut-liver-immune axis rather than merely suppressing its inflammatory outputs (55, 126–128).

### 6.3 Immune-directed therapy and precision endotyping

Two trafficking and myeloid-directed strategies illustrate both the promise and the present limits of immune-directed therapy. The CCR2/CCR5 antagonist cenicriviroc reduced inflammatory activity in the phase 2 CENTAUR trial but failed to demonstrate durable antifibrotic benefit in the subsequent phase 3 AURORA program, which was terminated for futility that underscoring that anti-inflammatory effect does not guarantee fibrosis reversal (133). In the PSC-IBD setting, the anti-α4β7 integrin antibody vedolizumab has shown heterogeneous effects on hepatic biochemistry despite controlling intestinal inflammation, consistent with the model that gut-imprinted lymphocyte homing to the liver may become partially autonomous once portal/biliary inflammation is established (134). Immune-directed therapy remains both promising and challenging. Blocking inflammatory trafficking pathways, reprogramming macrophage states, restoring antifibrotic or antitumor lymphocyte functions, or correcting MAIT cell and NKT cell dysfunction are all mechanistically attractive ideas, but overly broad immune manipulation can impair host defense in patients who are already prone to infection (9, 14, 64, 66). The balance between suppressing damaging inflammation and preserving antimicrobial competence is especially delicate in cirrhosis and alcoholic hepatitis. This is why precision endotyping is likely to be more useful than diagnosis-only treatment frameworks. Patients with the same clinical label may be driven predominantly by dysbiosis, BA dysregulation, barrier failure, immune trafficking, or toxin-producing pathobionts. Combining microbiome, metabolite, barrier, and immune-cell profiling may therefore be necessary to identify which node of the gut-liver-immune axis is dominant in a given patient (4, 5, 15). Endotyping should be coupled to disease stage, because the most relevant target shifts as disease advances. In early disease, steatosis, compensated MASLD/ALD, or pre-cirrhotic stages, the dominant and most reversible abnormalities are upstream: dysbiosis, barrier (epithelial and GVB) failure, and depletion of protective metabolites. Here the rational targets are barrier- and microbiota-directed: SCFA/butyrate and indole/AHR support, GVB stabilization, FXR-mediated BA normalization, mucus restoration, and selective pathobiont control, all of which aim to interrupt the axis before fixed tissue injury accrues. In late disease, advanced fibrosis, cirrhosis, decompensation, or established HCC, upstream correction alone is unlikely to reverse structural disease, and the relevant targets shift downstream to the immune and fibrotic compartments: macrophage reprogramming, trafficking inhibition, antifibrotic strategies, and, in HCC, checkpoint immunotherapy, while microbiota-directed measures retain value chiefly for complication-specific endpoints (e.g., rifaximin/FMT for HE) rather than disease reversal. The key caveat is reversibility: barrier and metabolite interventions are most likely to modify trajectory when the downstream niche is still intact, whereas in advanced disease the same interventions may improve selected endpoints without altering the fibrotic or oncogenic course (4, 5, 15, 22, 33, 63, 92–95, 114–118). Such stratification will also be essential for future biomarker development and for rational trial design in microbiota-targeted or immunomodulatory therapies.

### 6.4 Unresolved questions

Several key questions remain unresolved. First, causality is still difficult to prove in humans because diet, drugs, genetics, alcohol exposure, comorbidities, and disease stage all influence the microbiome and immune state. Second, there is important spatial and temporal heterogeneity: tissue-resident and recruited immune cells with similar surface markers can play very different roles depending on localization and stage, and longitudinal sampling of both gut and liver remains limited. Third, much of the strongest mechanistic work comes from animal models or simplified systems, which may not faithfully reproduce the complexity of human disease (4–6, 9). For immunology specifically, the axis shows why tissue context is indispensable. The same receptor, ligand, or cell subset can acquire different meaning depending on portal anatomy, BA state, stromal cues, and microbial ecology. Reviewing liver disease through this lens clarifies why translation has been difficult and where the most informative therapeutic opportunities may lie. The gut-liver-immune axis is one of the most mature frameworks in which to attempt that synthesis, and it is likely to remain a major conceptual engine for hepatology and mucosal immunology in the coming years (1–8).

**Figure 5:**
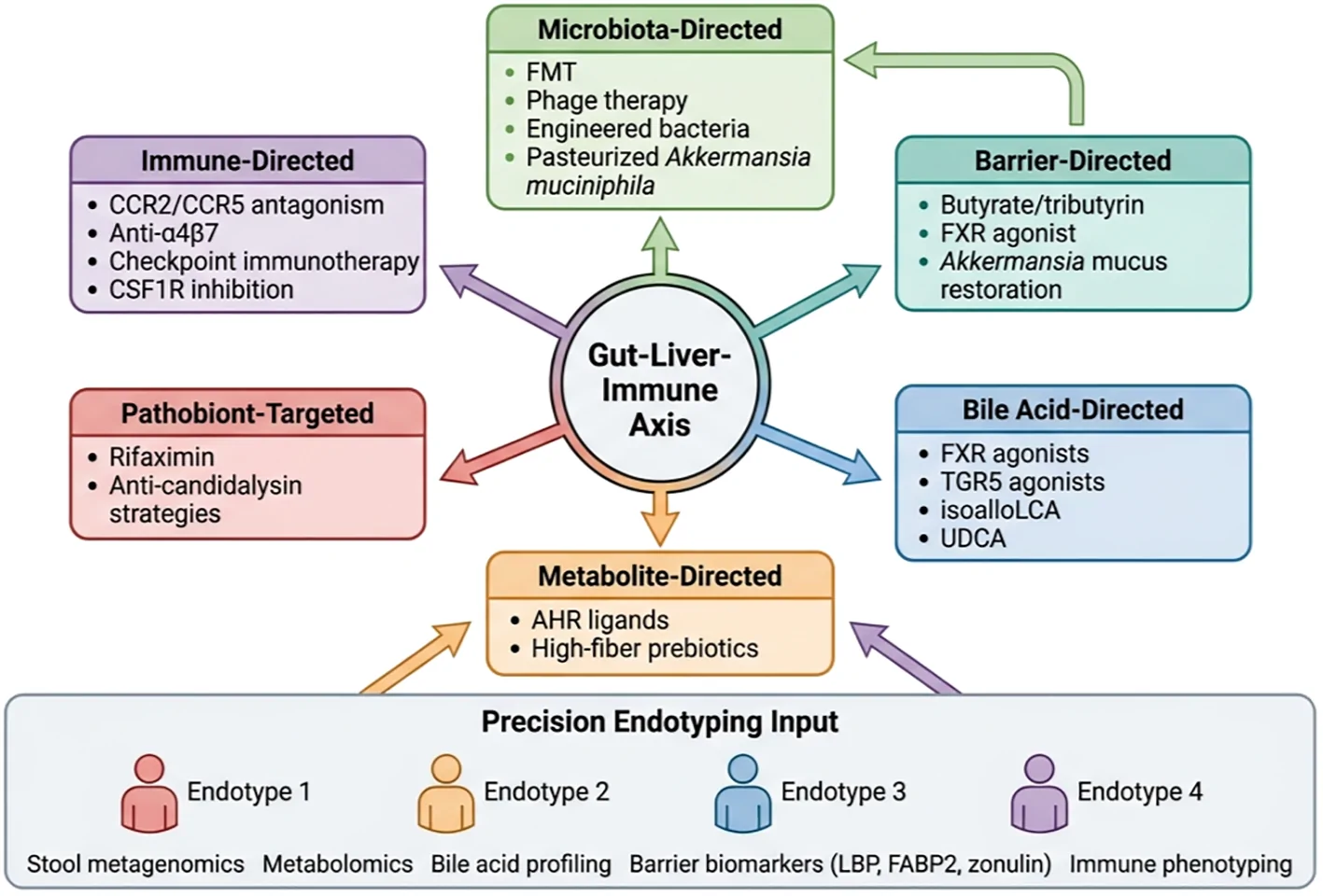
Therapeutic interception points across the gut-liver-immune axis: a precision endotype framework. Six intervention modules are arranged around a central gut-liver-immune axis schematic (1). Microbiota-directed: FMT with RCT evidence in ALD and cirrhosis (flagged for MDR transmission risk); phage therapy targeting cytolysin-positive *E. faecalis*; IL-22-secreting engineered bacteria and pasteurized *Akkermansia muciniphila* (Phase I) (2). Barrier-directed: butyrate/tributyrin (tight-junction reinforcement via HDAC inhibition); FXR agonist-mediated GVB stabilization (OCA, cilofexor); *Akkermansia* mucus restoration (3). BA-directed: FXR agonists (OCA, cilofexor, tropifexor: FGF19 induction and CYP7A1 suppression); TGR5 agonists (macrophage NF-κB suppression via cAMP-PKA, GLP-1 release); isoalloLCA (Treg induction); UDCA (cholangiocyte cytoprotection) (4). Metabolite-directed: AHR ligands including indole, IAA, and IPA (restoring IL-22 and tight-junction gene expression); high-fiber prebiotic diets (SCFA and Treg-inducing pathway enrichment via GPR43/GPR109A) (5). Pathobiont-targeted: rifaximin (selective intestinal decontamination); anti-candidalysin neutralization (6). Immune-directed: CCR2/CCR5 antagonism (cenicriviroc); anti-α4β7 (vedolizumab) or anti-CCR9 in PSC; anti-PD-1/PD-L1 checkpoint immunotherapy in HCC; CSF1R inhibition. The precision endotyping panel at the base integrates stool metagenomics, serum metabolomics, BA profiling, barrier biomarkers (LBP, FABP2, zonulin), and immune phenotyping to classify patients into dominant endotypes and assign the most relevant intervention module, with longitudinal endpoints including microbiome diversity index, serum FGF19, intestinal permeability markers, and liver stiffness by VCTE. AHR, aryl hydrocarbon receptor; ALD, alcohol-associated liver disease; BA, bile acid; cAMP, cyclic adenosine monophosphate; CCR, C-C chemokine receptor; CSF1R, colony stimulating factor 1 receptor; CYP7A1, Cholesterol 7α-Hydroxylase; FABP2, fatty acid binding protein 2; FGF19, fibroblast growth factor 19 (human); FMT, fecal microbiota transplantation; FXR, farnesoid X receptor; GLP-1, glucagon-like peptide-1; GPR, G-protein coupled receptors; GVB, gut vascular barrier; HCC, hepatocellular carcinoma; HDAC, histone deacetylase; IAA, indole-3-acetic acid; IL, interleukin; IPA, indole-3-propionic acid; isoalloLCA, isoallolithocholic acid; LBP, LPS-binding protein; MDR, multidrug-resistant; NF-κB, nuclear factor kappa B; OCA, obeticholic acid; PD-1, programmed cell death protein 1; PD-L1, programmed death ligand 1; PKA, protein kinase A; PSC, primary sclerosing cholangitis; RCT, randomized controlled trial; SCFA, short-chain fatty acid; TGR5, Takeda G-protein coupled receptor 5; Treg, regulatory T cell; UDCA, ursodeoxycholic acid; VCTE, vibration-controlled transient elastography.

It is worth stating explicitly which experimental platforms are best matched to which gaps. The problem of causal inference in humans is most tractable in patient-derived intestinal and hepatic organoids and in gut-liver microphysiological systems (MPS/organ-on-chip), which permit controlled, reductionist interrogation of barrier–metabolite–immune interactions with human cells and have already revealed non-intuitive, sometimes paradoxical, metabolite effects on inter-organ inflammation (135). The problem of spatial and temporal heterogeneity is best addressed by single-cell and spatial transcriptomic atlases, which can localize resident-versus-recruited populations within zonated niches, as recent PSC and fibrotic-liver atlases demonstrate (136, 137). The problem of host–microbe causality at the organismal level is the domain of gnotobiotic and humanized-microbiota models, with the interpretive caveats detailed in section 7.5 Limitations part. No single platform is sufficient; the most convincing future work will triangulate organoid/MPS mechanism, spatial-omic localization, and *in vivo* transmissibility against carefully phenotyped human cohorts (4–6, 9, 136–140) (**Figure 5**, **Table 4**).

### 6.5 Limitations

Several limitations constrain the strength of the conclusions synthesized here and should guide cautious interpretation. First, much of the mechanistic evidence derives from rodent models that gnotobiotic, germ-free, and humanized-microbiota systems, whose microbial ecology, bile acid pool composition (e.g., the prominence of muricholic acids in mice), and immune architecture differ materially from those of humans, so that demonstrations of transmissibility or pathway dependence in mice do not establish human causality. The bile acid axis offers the clearest illustration: mice synthesize abundant muricholic acids that act as endogenous FXR antagonists, giving the murine BA pool hydrophilicity and receptor-signaling profile fundamentally different from the human pool, so that FXR-dependent phenotypes observed in mice cannot be assumed to scale to patients (100). More broadly, human-microbiota-associated rodents, frequently used to argue for causality, reconstitute only a fraction of the donor community and impose a murine host context on human microbes, such that engraftment artifacts and host-species mismatch can generate phenotypes that overstate or distort the human relationship (141). These considerations argue for treating murine mechanism as hypothesis-generating and reserving causal claims for findings corroborated in human tissue, human organoid/MPS systems, or prospective clinical data. Second, a large fraction of the human data is cross-sectional and associative; reverse causation is plausible because hepatic dysfunction, altered portal hemodynamics, medications, and diet all reshape the microbiome and metabolome independently of any primary microbial driver. Third, peripheral blood immunophenotyping frequently diverges from tissue-resident biology, and longitudinal paired gut–liver sampling in humans remains scarce, limiting inference about resident-versus-recruited immune populations. Fourth, barrier dysfunction is heterogeneous and is often inferred from indirect surrogates (zonulin, LBP, FABP2) whose specificity is debated, rather than from direct regional permeability measurement. Finally, microbiota- and immune-directed interventions show substantial inter-individual variability in engraftment and response, and several have not advanced beyond phase 2, so their translational generalizability is not yet established. These limitations motivate the longitudinal, multi-omic, tissue-aware study designs outlined below.

## 7 Conceptual synthesis and future perspectives

### 7.1 Why the gut-liver-immune axis matters beyond hepatology

A reason to take the gut-liver-immune axis seriously is that it likely models a broader class of inter-organ immune circuits. The liver receives constant environmental information from the intestine, converts much of that information into metabolic and immune output, and then redistributes signals back to the intestine and the systemic circulation. This makes the liver not only a target organ but also a central interpreter of host-microbe relationships. In that sense, gut-liver biology may provide general lessons for gut-adipose, gut-brain, and gut-kidney communication, particularly where immune cells serve as mobile effectors of tissue crosstalk (1–3, 7). Therapeutic translation should also be framed in terms of endotypes rather than disease labels. A precision framework would therefore combine microbial composition, metabolite state, host genetics, immune-cell distribution, and clinical stage to identify the most tractable node of the axis in each patient. Such an approach is more complex than diagnosis-based treatment algorithms, but it is probably the way to exploit the full therapeutic potential of the gut-liver-immune concept (2, 3, 7, 8, 43, 102, 107).

### 7.2 Translational design principles for the next decade

Several practical design principles emerge from the current literature. First, future studies should pair ecological readouts with host response readouts; stool sequencing without immune phenotyping, metabolomics, or barrier assessment is insufficient for understanding axis biology. Second, disease stage matters. An intervention that is beneficial in steatosis or compensated cirrhosis may fail or even become harmful in alcoholic hepatitis or AD, where antimicrobial competence is already compromised (75, 98, 99). Third, longitudinal sampling is more informative than cross-sectional sampling because many of the most important events in the gut-liver-immune axis are dynamic rather than static, including trafficking, BA remodeling, and intermittent translocation. Fourth, careful attention to niche biology is needed, because peripheral blood measurements often underrepresent tissue processes in the liver and intestine (6, 7, 67–69). Even in the strongest mechanistic examples, causality remains layered. The real task is therefore not to prove that one organism or pathway causes liver disease in a simple sense, but to define the conditions under which a given microbial or immune signal becomes decisive for disease progression. Mechanistic precision of that kind will be necessary if microbiota-directed therapies are to move beyond empiricism and into rational patient selection (2, 3, 8, 43, 75, 99, 107).

### 7.3 From association to mechanism: what constitutes convincing causality?

Because microbiome studies have multiplied rapidly, the field now faces a high bar for causal inference. Mere association between a microbial signature and disease severity is no longer sufficient. Convincing mechanistic evidence now usually requires one or more of the following: transmissibility in model systems, identification of a bioactive metabolite or toxin, demonstration of a host receptor or immune pathway, validation in tissue-relevant models, and some degree of concordance with human clinical data. Several exemplary studies meet much of this standard, including work on inflammasome-mediated dysbiosis in MASLD, cytolysin-positive *Enterococcus* in ALD, candidalysin in alcohol-associated liver injury, BA metabolite effects on Treg/Th17 balance, and microbiota-dependent control of NKT-mediated tumor surveillance (53–57, 70, 72–74, 78, 91–93, 111). These studies should serve as templates for future mechanistic and translational work.

### 7.4 Cholangiocytes, portal structures, and the neglected biliary interface

Many discussions of the gut-liver axis remain hepatocyte centered, yet the biliary tree is itself a major immunological and signaling interface. Cholangiocytes are directly exposed to BAs and biliary metabolites, respond to inflammatory mediators, and sit anatomically close to portal tracts where incoming immune cells are concentrated. This is especially relevant to PSC, in which portal inflammation, cholangiocyte stress, endothelial activation, and gut-imprinted lymphocyte recruitment coexist (11, 12, 79–82). From a gut-liver-immune perspective, cholangiopathy may represent a disease of distorted information flow: abnormal bile composition reshapes the intestine, intestinal inflammation generates misdirected immune programs, and portal and biliary structures in the liver receive both forms of abnormal signaling. This view suggests that cholangiocyte biology should be integrated more directly into future models of inter-organ immunity rather than treated as a purely downstream target of inflammation. The biliary interface is also conceptually important because it demonstrates that liver-to-gut communication is not limited to classical BA recycling. Biliary flow can influence microbial ecology, barrier tone, and antigen availability; conversely, changes in the intestinal ecosystem alter the pool of secondary BAs and other metabolites that return to the biliary-hepatic compartment (1–3, 7, 25–30, 61, 63). The portobiliary axis may therefore be a particularly powerful site for biomarker development, especially in cholestatic and autoimmune liver diseases. Although accessible human sampling remains a challenge, studies that integrate stool, serum, bile, and tissue immune profiling could clarify whether specific biliary metabolite states correspond to particular immune endotypes. The practical limitations of human bile sampling deserve explicit acknowledgment, because they constrain how the portobiliary axis can realistically be studied. Direct bile collection in humans generally requires invasive access, endoscopic retrograde cholangiopancreatography (ERCP), percutaneous or surgical biliary drains, or intraoperative aspiration, each carrying procedural risk (e.g., post-ERCP pancreatitis), selection bias toward patients with biliary pathology, and an almost complete absence of healthy-control bile for comparison. Sampled bile is also prone to contamination from instrumentation and to compositional change with cholestasis or drainage, complicating interpretation. For these reasons, validated non-invasive surrogates are needed. Candidate surrogates include serum bile acid profiles, which partially reflect the enterohepatic and biliary BA pool, and serum FGF19 as a readout of intestinal FXR activation and enterohepatic BA signaling (142); fecal bile acid quantification as an integrated readout of microbial BA transformation; and circulating barrier and cholestatic markers. Emerging approaches, bile- or serum-derived extracellular vesicles, metabolomic signatures, and functional imaging, may further reduce reliance on direct aspiration. We propose that integrated stool–serum–bile profiling be pursued where bile is available for clinical reasons (e.g., during ERCP or transplantation), while non-invasive BA/FGF19-based surrogates are developed and validated for broader use, particularly in cholestatic and autoimmune liver diseases where the portobiliary interface is most relevant (3, 7, 25–27, 61, 63). That type of integrated sampling may ultimately be more informative than fecal composition alone, especially when disease biology is localized to portal tracts rather than diffuse parenchyma.

### 7.5 Immune-cell plasticity, tissue residency, and replacement dynamics

A key challenge for the field is understanding the extent to which lymphocyte behavior within the gut-liver-immune axis is stable, adaptive, or pathologically fixed. MAIT cells, NKT cells, NK cells, and adaptive T-cell subsets all display strong context dependence and may transition from antimicrobial or regulatory roles toward profibrotic or dysfunctional states under chronic stimulation (18, 65–69, 111). Their behavior in chronic liver disease reflects a fundamental tension between the persistence of tissue residency programs and the disruption of normal niche cues by sustained inflammation, metabolic dysregulation, or barrier failure. Circulating measurements often suggest depletion, whereas tissue studies suggest local accumulation, altered residency, or activation. This gap between blood and tissue observations is a major translational obstacle. For diseases such as PSC, in which tissue-specific homing is a central feature, blood phenotyping alone can be actively misleading (11, 12, 79–82). A more precise model will therefore require longitudinal, tissue-aware immunology that can distinguish resident from recruited cells and define when a cell population is protective, exhausted, pathogenic, or reparative. Single-cell and spatial platforms are likely to be especially valuable here, but their interpretation will depend on careful integration with barrier and metabolite data (6, 7, 136–140).

Another unresolved issue is how stable versus replaceable the immune architecture of the axis is. Macrophage biology illustrates the problem particularly well. Homeostatic Kupffer cells, recruited monocyte-derived macrophages, and intestinal macrophage subsets may share some markers while remaining functionally distinct because of divergent developmental origin, niche cues, and metabolic context (5, 17, 38, 67). During chronic disease, incoming monocytes can reshape the liver far beyond a transient inflammatory burst by changing cytokine gradients, antigen presentation, fibrogenic signaling, and interactions with endothelial and stellate cells. At the same time, intestinal macrophages influence epithelial repair and permeability, meaning that myeloid cells can simultaneously worsen liver injury and weaken the upstream barrier that feeds the injury. This bidirectional myeloid pathology is one reason macrophage-directed therapies are attractive yet difficult to design: simply removing inflammatory cells may also eliminate cells required for repair, microbial containment, or resolution (4, 5, 8).

### 7.6 Spatial organization and immune zonation across the axis

A recurring theme in recent work is that the gut-liver-immune axis is spatially organized rather than diffuse. In the liver, blood flow from portal tracts to central veins generates gradients of oxygen, nutrients, microbial products, BA intermediates, and morphogens, and these gradients are translated into zonated programs in both parenchymal and non-parenchymal cells. Spatial studies have shown that immune surveillance, endothelial behavior, and macrophage positioning are also anatomically patterned, implying that the same circulating signal may be interpreted differently depending on where it arrives within the lobule (23, 136–140). Hepatic immune zonation driven by commensals suggests that microbial influence on the liver is anatomically patterned even in health, and that this organization probably changes with steatosis, cholestasis, fibrosis, and cirrhosis (3, 17, 26, 31, 41).

Spatial organization also matters on the intestinal side of the axis. The small intestine, colon, Peyer’s patches, mucus layers, GVB, and mesenteric immune structures all receive and process different antigenic and metabolic inputs. IgA-secreting cells originating from Peyer’s patches can be found in the liver, and BA concentrations and microbial density vary along the intestine, meaning that FXR-driven and TGR5-driven programs are likely to differ between segments and disease contexts (7, 18, 22, 28–31, 33). These segmental differences are mechanistically consequential for the hepatic niche rather than merely descriptive. The proximal small intestine, and the ileum in particular, is the dominant site of FXR-mediated BA sensing and of FGF15/FGF19 induction; ileal signals therefore preferentially set the hepatic BA-synthesis rate (via CYP7A1 repression) and condition periportal myeloid tone. The colon, by contrast, is the principal site of fiber fermentation to SCFAs and of microbial 7α-dehydroxylation to secondary BAs (DCA, LCA), so distal signals preferentially shape Treg/Th17 balance and macrophage imprinting. Because portal blood integrates inputs from all segments but with segment-specific metabolite spectra, the hepatic immune niche effectively receives a regionally weighted signal whose composition shifts with the anatomical site of barrier failure, proximal barrier failure biasing toward BA/FGF-axis dysregulation and distal barrier failure toward SCFA depletion and secondary BA-dependent immune skewing. This regional logic mirrors the established concept of compartmentalized, segment-specific specialization within the intestinal immune system itself (143), and it implies that where the barrier fails may be as informative as whether it fails (7, 18, 22, 28–31, 33). A major challenge for the next phase of the field is therefore to move beyond the binary language of leaky gut versus intact gut and toward a finer cartography of where barrier failure occurs, which molecules cross, and which immune niches are first engaged (3, 8, 43, 75, 99, 102). Recent spatial and single-cell atlases further suggest that disease disrupts these ordered niches in a lesion-specific manner. In PSC, spatial transcriptomics has identified scar-associated cholangiocyte-like hepatocytes, chemokine-rich mesenchymal and endothelial compartments, and recruited immune populations concentrated around biliary lesions rather than distributed uniformly (136). In fibrotic human liver, high-resolution spatial mapping shows loss of normal zonal architecture together with remodeling of receptor-ligand neighborhoods involving macrophages, mesenchymal cells, and hepatocytes (137). These observations argue that future gut-liver studies should treat zonation as a mechanistic variable rather than a histological curiosity (136–140).

### 7.7 Integrating ecology, metabolism, and immunity in clinical interpretation

The field should remain cautious about assuming that normalization of the microbiota is always the desired endpoint. Restoring one taxonomic pattern may not restore the underlying immune ecology if epithelial repair, BA signaling, or tissue-resident immune dysfunction remain uncorrected. Conversely, some therapies may improve immune tone and liver outcomes without producing dramatic changes in overall microbial composition. This systems-level interconnection also defines the principal trade-offs of pathway-specific intervention. Because the nodes of the axis are coupled, a manipulation that corrects one node frequently perturbs others: systemic FXR agonism normalizes hepatic BA synthesis but simultaneously reshapes the intestinal BA pool, microbial ecology, and lipid handling, with off-target dyslipidemia; broad macrophage depletion or trafficking blockade may reduce damaging inflammation while impairing antimicrobial defense and resolution in hosts already prone to infection; and aggressive microbiota ablation (e.g., non-selective antibiotics) can deplete protective commensals along with pathobionts. Pathway specificity is therefore rarely clean, and the relevant question is whether the intended on-target benefit outweighs predictable perturbation of interconnected systems in a given disease stage. A related question is why protective pathways fail in disease and whether they can be reliably restored. Failure modes are heterogeneous: substrate depletion (loss of SCFA- or indole-producing commensals removes the input to GPR43/AHR signaling), receptor-level changes (FXR desensitization or altered expression), niche disruption (loss of the metabolic and stromal cues that sustain tissue-resident regulatory cells), and frank irreversibility (established fibrosis or senescence-associated remodeling). These modes have different restorability: input-limited failures (e.g., SCFA/indole depletion) are the most plausibly correctable by metabolite or microbial replacement, provided the downstream receptor and niche remain intact, whereas niche-disrupted or structurally irreversible failures are unlikely to respond to upstream replacement alone. Reliable restoration therefore requires matching the intervention to the failure mode, and confirming, ideally with tissue-level readouts, that the protective program is still inducible rather than assuming that supplying a metabolite will reconstitute a lost function (25, 63, 73, 94). This possibility is already suggested by work on metabolites, postbiotics, engineered bacteria, and host-targeted BA signaling (25, 63, 127). A mature translational agenda will therefore need outcome measures that capture function, not just composition.

The integrative approach is especially important in HCC surveillance and in the management of compensated cirrhosis. Microbiota profiles associated with HCC, decompensation, or HE probably capture more than microbial abundance; they may also reflect impaired barrier architecture, altered BA circulation, and immune exhaustion or activation states that are not directly visible in stool sequencing alone (96, 119, 121). A central practical consequence of this framework is that clinical interpretation should integrate ecology, metabolism, and immunity rather than treating them as separate readouts. A microbial signature associated with fibrosis, decompensation, or HCC may represent an upstream driver in one patient but a secondary consequence of altered BA flow, portal hemodynamics, medications, or immune dysfunction in another. To make this distinction operational, we propose explicit criteria for separating a driver from a marker. A microbial taxon or metabolite qualifies as a candidate driver when it satisfies several of the following: demonstrable transmissibility of the phenotype (e.g., by colonization or FMT in model systems), identification of a defined bioactive species (toxin or metabolite) and its host receptor or immune pathway, a plausible dose–response relationship, and corroboration across human tissue and mechanistically anchored models. A signal that fluctuates with disease activity but fails these tests, particularly one fully explained by altered bile flow, reduced hepatic clearance, diet, or medication, is better classified as a marker. Host factors are decisive in this assignment: host genetics (e.g., variants affecting BA transport, innate sensing, or lipid handling), baseline hepatic function, intestinal transit, diet, and concomitant drugs all determine whether a given microbial output is generated, sensed, and translated into immune activation. The same metabolite can therefore be a driver in a permissive host background and an inert marker in another, which is precisely why composition-only readouts are insufficient and why driver-versus-marker classification must be made at the level of the individual host–microbe configuration rather than the population average (4, 5, 66, 70, 144). Metabolite profiles cannot be interpreted in isolation, because BAs, short-chain fatty acids, indole derivatives, and endogenous ethanol are shaped simultaneously by diet, microbial ecology, epithelial transport, liver function, and host inflammatory tone. The strongest future biomarkers will likely integrate microbial, metabolic, barrier-based, and immune information into a single mechanistic readout rather than treating these domains as separate data silos (4, 5, 63, 144).

Emerging biomarker studies illustrate both the promise and the limitation of reductionist interpretation. Multi-omic analyses in cirrhosis have identified complication-associated microbial and metabolite signatures, while metagenomic models have shown diagnostic utility for cirrhosis itself and prognostic utility for hepatic decompensation and mortality in MASLD-related cirrhosis (144–146). Going forward, clinically useful models of the gut-liver-immune axis will need longitudinal and multimodal design. Stool metagenomics, fecal and serum metabolomics, BA profiling, inflammatory mediators, and carefully phenotyped immune-cell measurements should be interpreted together and tracked across disease stage, treatment exposure, and decompensating events (13, 131, 132, 144–146).

The evidence synthesized in this review supports a shift from the classical gut-liver axis to a broader gut-liver-immune axis. This expanded model better reflects the reality that inter-organ communication is not governed by microbial composition or metabolite abundance alone, but by the way those inputs are filtered through epithelial and vascular barriers, interpreted by tissue-resident and recruited immune cells, and reshaped by BA signaling, hepatobiliary function, and disease stage. In health, these interacting layers support tolerance, nutrient handling, antimicrobial defense, and controlled tissue repair. In disease, the same circuits can become destabilized, allowing dysbiosis, barrier failure, aberrant trafficking, and maladaptive immune-cell activation to drive steatohepatitis, alcohol-related injury, cholangiopathy, cirrhosis, decompensation, and HCC (3–5, 8, 13, 14).

A central message is that immune cells are not secondary bystanders within the axis. Macrophages, DCs, neutrophils, MAIT cells, NKT cells, NK cells, and adaptive lymphocyte subsets act as mobile and context-sensitive integrators that convert ecological and metabolic signals into tissue outcomes. Their behavior depends on developmental origin, spatial niche, local metabolites, and temporal stage of injury, which helps explain why similar microbial perturbations can produce different clinical phenotypes and why blood-based immune measurements often fail to capture the biology of the liver itself (5, 14, 17, 136, 137).

**Figure 6:**
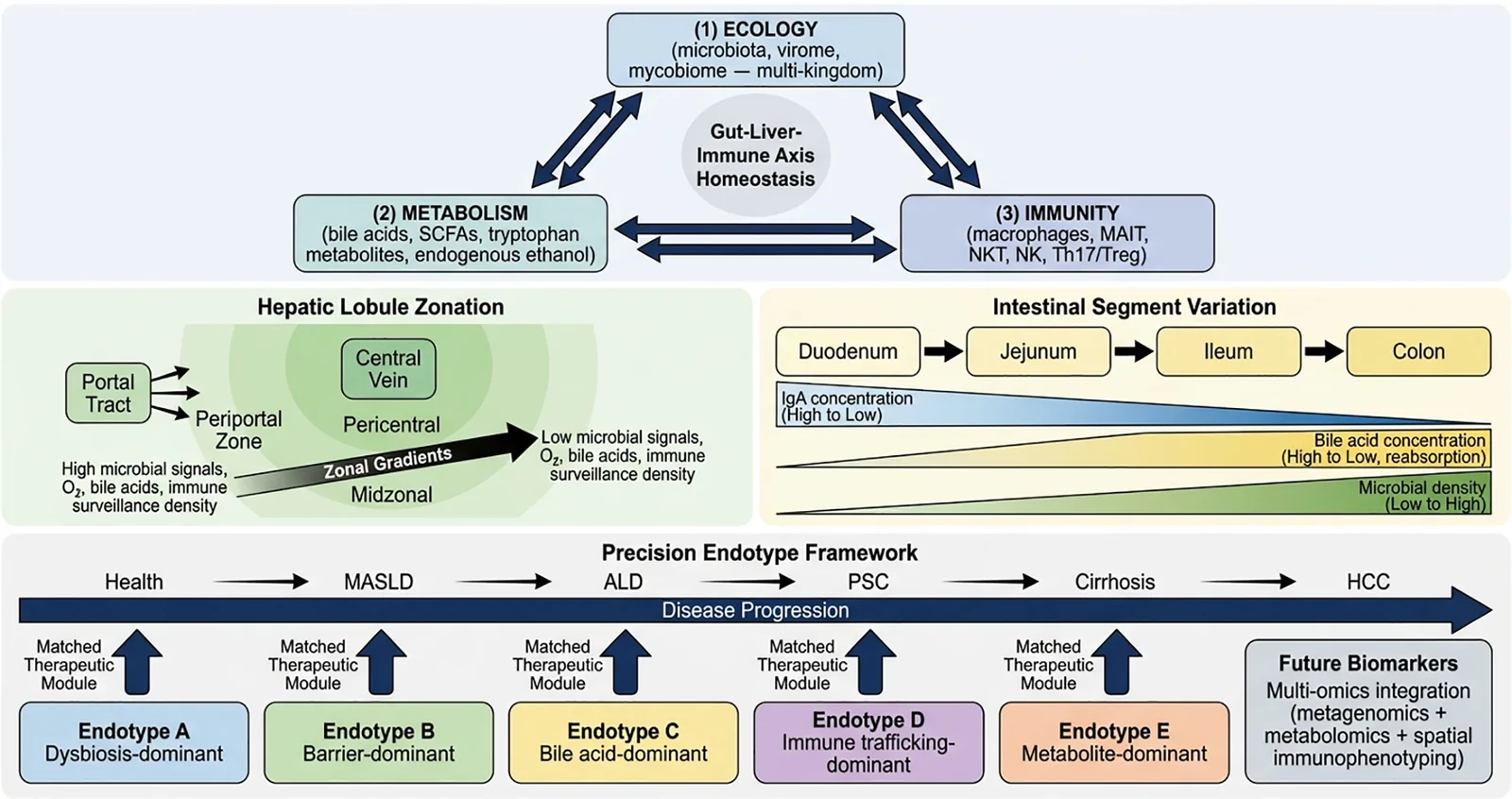
Integrative conceptual framework of the gut-liver-immune axis and precision endotyping strategy. Top panel (Triadic Relationship): an equilateral triangle illustrating the bidirectional interactions among the three core axes of the gut-liver-immune system, Ecology (multi-kingdom microbiota: bacteria, virome, mycobiome), Metabolism (BAs, SCFAs, tryptophan metabolites, endogenous ethanol), and Immunity (macrophages, MAIT/NKT/NK cells, Th17/Treg), with bidirectional labeled arrows between all three nodes and the label “Gut-Liver-Immune Axis Homeostasis” at center. Middle panel (Spatial Organization): two complementary schematics showing hepatic lobular zonation (gradients of oxygen, microbial products, BA intermediates, and immune surveillance density from portal tract to central vein) and intestinal segmental variation (BA concentration, microbial density, and FXR/TGR5 program distribution from duodenum to colon), illustrating that the axis is spatially organized rather than diffuse. Bottom panel (Precision Endotype Framework): a horizontal disease progression arrow from Health through MASLD, ALD, PSC, Cirrhosis, to HCC, overlaid with five color-coded precision endotypes. **(A)** Dysbiosis-dominant, **(B)** Barrier-dominant, **(C)** BA-dominant, **(D)** Immune trafficking-dominant, and **(E)** Metabolite-dominant, each linked by upward arrows to its matched therapeutic module, with multi-omics biomarker integration (metagenomics + metabolomics + spatial immunophenotyping) shown as the foundation for endotype-informed clinical decision-making. ALD, alcohol-associated liver disease; BA, bile acid; FXR, farnesoid X receptor; HCC, hepatocellular carcinoma; MAIT, mucosal-associated invariant T cell; MASLD, metabolic dysfunction-associated steatotic liver disease; NK, natural killer; NKT, natural killer T cell; PSC, primary sclerosing cholangitis; SCFA, short-chain fatty acid; TGR5, Takeda G-protein coupled receptor 5; Treg, regulatory T cell.

Finally, translation will depend on moving beyond single-layer descriptions of the microbiome or isolated inflammatory markers. The most informative future studies will combine ecology, metabolism, spatial tissue biology, and immunophenotyping in longitudinal human cohorts and mechanistically anchored models. Such integration should improve biomarker interpretation, refine disease endotyping, and help identify the patients most likely to benefit from barrier-protective strategies, BA modulation, microbiota-directed therapy, or immune-targeted treatment. The gut-liver-immune axis is therefore not just a new label for an existing field; it is a more precise framework for explaining heterogeneity across liver diseases and for designing the next generation of diagnostic and therapeutic strategies (144–146) (**Figure 6**).

## References

[1] Marcellin P Kutala BK . Liver diseases: a major, neglected global public health problem requiring urgent actions and large-scale screening. Liver Int. (2018) 38:2–6. doi: 10.1111/liv.13682 29427496

[2] Asrani SK Devarbhavi H Eaton J Kamath PS . Burden of liver diseases in the world. J Hepatol. (2019) 70:151–71. doi: 10.1016/j.jhep.2018.09.014 30266282

[3] Pabst O Hornef MW Schaap FG Cerovic V Clavel T Bruns T . Gut-liver axis: barriers and functional circuits. Nat Rev Gastroenterol Hepatol. (2023) 20:447–61. doi: 10.1038/s41575-023-00771-6 37085614

[4] Hsu CL Schnabl B . The gut-liver axis and gut microbiota in health and liver disease. Nat Rev Microbiol. (2023) 21:719–33. doi: 10.1038/s41579-023-00904-3 37316582 PMC10794111

[5] Tilg H Adolph TE Trauner M . Gut-liver axis: pathophysiological concepts and clinical implications. Cell Metab. (2022) 34:1700–18. doi: 10.1016/j.cmet.2022.09.017 36208625

[6] Albillos A de Gottardi A Rescigno M . The gut-liver axis in liver disease: pathophysiological basis for therapy. J Hepatol. (2020) 72:558–77. doi: 10.1016/j.jhep.2019.10.003 31622696

[7] Tripathi A Debelius J Brenner DA Karin M Loomba R Schnabl B . The gut-liver axis and the intersection with the microbiome. Nat Rev Gastroenterol Hepatol. (2018) 15:397–411. doi: 10.1038/s41575-018-0011-z 29748586 PMC6319369

[8] Bruneau A Hundertmark J Guillot A Tacke F . Molecular and cellular mediators of the gut-liver axis in the progression of liver diseases. Front Med (Lausanne). (2021) 8:725390. doi: 10.3389/fmed.2021.725390 34650994 PMC8505679

[9] De Muynck K Vanderborght B Van Vlierberghe H Devisscher L . The gut-liver axis in chronic liver disease: a macrophage perspective. Cells. (2021) 10. doi: 10.3390/cells10112959 34831182 PMC8616442

[10] Szabo G . Gut-liver axis in alcoholic liver disease. Gastroenterology. (2015) 148:30–6. doi: 10.1053/j.gastro.2014.10.042 25447847 PMC4274189

[11] Tabibian JH O'Hara SP Larusso NF . Primary sclerosing cholangitis: the gut-liver axis. Clin Gastroenterol Hepatol. (2012) 10:819–20. doi: 10.1016/j.cgh.2012.01.024 22343691

[12] Karlsen TH Folseraas T Thorburn D Vesterhus M . Primary sclerosing cholangitis - a comprehensive review. J Hepatol. (2017) 67:1298–323. doi: 10.1016/j.jhep.2017.07.022 28802875

[13] Trebicka J Macnaughtan J Schnabl B Shawcross DL Bajaj JS . The microbiota in cirrhosis and its role in hepatic decompensation. J Hepatol. (2021) 75 Suppl 1:S67–81. doi: 10.1016/j.jhep.2020.11.013 34039493 PMC8973011

[14] Bozward AG Ronca V Osei-Bordom D Oo YH . Gut-liver immune traffic: deciphering immune-pathogenesis to underpin translational therapy. Front Immunol. (2021) 12:711217. doi: 10.3389/fimmu.2021.711217 34512631 PMC8425300

[15] Gu Y Wu C . The gut-liver axis modulates intestinal immune homeostasis. Mucosal Immunol. (2026) 19:1470–80. doi: 10.1016/j.mucimm.2025.12.001 41435890 PMC13321230

[16] Atif M Warner S Oo YH . Linking the gut and liver: crosstalk between regulatory T cells and mucosa-associated invariant T cells. Hepatol Int. (2018) 12:305–14. doi: 10.1007/s12072-018-9882-x 30027532 PMC6097019

[17] Gebru YA Choi MR Raja G Gupta H Sharma SP Choi YR . Pathophysiological roles of mucosal-associated invariant T cells in the context of gut microbiota-liver axis. Microorganisms. (2021) 9. doi: 10.3390/microorganisms9020296 33535703 PMC7912788

[18] Bolte FJ Rehermann B . Mucosal-associated invariant T cells in chronic inflammatory liver disease. Semin Liver Dis. (2018) 38:60–5. doi: 10.1055/s-0037-1621709 29471566 PMC6283052

[19] Borovikova LV Ivanova S Zhang M Yang H Botchkina GI Watkins LR . Vagus nerve stimulation attenuates the systemic inflammatory response to endotoxin. Nature. (2000) 405:458–62. doi: 10.1038/35013070 10839541

[20] Teratani T Mikami Y Nakamoto N Suzuki T Harada Y Okabayashi K . The liver-brain-gut neural arc maintains the T(Reg) cell niche in the gut. Nature. (2020) 585:591–6. doi: 10.1038/s41586-020-2425-3 32526765

[21] Balmer ML Slack E de Gottardi A Lawson MA Hapfelmeier S Miele L . The liver may act as a firewall mediating mutualism between the host and its gut commensal microbiota. Sci Transl Med. (2014) 6:237ra66. doi: 10.1126/scitranslmed.3008618 24848256

[22] Mouries J Brescia P Silvestri A Spadoni I Sorribas M Wiest R . Microbiota-driven gut vascular barrier disruption is a prerequisite for non-alcoholic steatohepatitis development. J Hepatol. (2019) 71:1216–28. doi: 10.1016/j.jhep.2019.08.005 31419514 PMC6880766

[23] Gola A Dorrington MG Speranza E Sala C Shih RM Radtke AJ . Commensal-driven immune zonation of the liver promotes host defence. Nature. (2021) 589:131–6. doi: 10.1038/s41586-020-2977-2 33239787 PMC8691525

[24] McDonald B Zucoloto AZ Yu IL Burkhard R Brown K Geuking MB . Programing of an intravascular immune firewall by the gut microbiota protects against pathogen dissemination during infection. Cell Host Microbe. (2020) 28:660–8. doi: 10.1016/j.chom.2020.07.014 32810440

[25] Fuchs CD Trauner M . Role of bile acids and their receptors in gastrointestinal and hepatic pathophysiology. Nat Rev Gastroenterol Hepatol. (2022) 19:432–50. doi: 10.1038/s41575-021-00566-7 35165436

[26] Inagaki T Choi M Moschetta A Peng L Cummins CL McDonald JG . Fibroblast growth factor 15 functions as an enterohepatic signal to regulate bile acid homeostasis. Cell Metab. (2005) 2:217–25. doi: 10.1016/j.cmet.2005.09.001 16213224

[27] Kliewer SA Mangelsdorf DJ . Bile acids as hormones: the FXR-FGF15/19 pathway. Dig Dis. (2015) 33:327–31. doi: 10.1159/000371670 26045265 PMC4465534

[28] Jakobsson HE Rodriguez-Pineiro AM Schutte A Ermund A Boysen P Bemark M . The composition of the gut microbiota shapes the colon mucus barrier. EMBO Rep. (2015) 16:164–77. doi: 10.15252/embr.201439263 25525071 PMC4328744

[29] Johansson ME Phillipson M Petersson J Velcich A Holm L Hansson GC . The inner of the two Muc2 mucin-dependent mucus layers in colon is devoid of bacteria. Proc Natl Acad Sci USA. (2008) 105:15064–9. doi: 10.1073/pnas.0803124105 18806221 PMC2567493

[30] Vaishnava S Yamamoto M Severson KM Ruhn KA Yu X Koren O . The antibacterial lectin Regiiigamma promotes the spatial segregation of microbiota and host in the intestine. Science. (2011) 334:255–8. doi: 10.1126/science.1209791 21998396 PMC3321924

[31] Bunker JJ Bendelac A . IgA responses to microbiota. Immunity. (2018) 49:211–24. doi: 10.1016/j.immuni.2018.08.011 30134201 PMC6107312

[32] Spadoni I Zagato E Bertocchi A Paolinelli R Hot E Di Sabatino A . A gut-vascular barrier controls the systemic dissemination of bacteria. Science. (2015) 350:830–4. doi: 10.1126/science.aad0135 26564856

[33] Sorribas M Jakob MO Yilmaz B Li H Stutz D Noser Y . FXR modulates the gut-vascular barrier by regulating the entry sites for bacterial translocation in experimental cirrhosis. J Hepatol. (2019) 71:1126–40. doi: 10.1016/j.jhep.2019.06.017 31295531

[34] Hooper LV Midtvedt T Gordon JI . How host-microbial interactions shape the nutrient environment of the mammalian intestine. Annu Rev Nutr. (2002) 22:283–307. doi: 10.1146/annurev.nutr.22.011602.092259 12055347

[35] Lee JY Tsolis RM Baumler AJ . The microbiome and gut homeostasis. Science. (2022) 377:eabp9960. doi: 10.1126/science.abp9960 35771903

[36] Schulthess J Pandey S Capitani M Rue-Albrecht KC Arnold I Franchini F . The short chain fatty acid butyrate imprints an antimicrobial program in macrophages. Immunity. (2019) 50:432–45. doi: 10.1016/j.immuni.2018.12.018 30683619 PMC6382411

[37] Arpaia N Campbell C Fan X Dikiy S van der Veeken J deRoos P . Metabolites produced by commensal bacteria promote peripheral regulatory T-cell generation. Nature. (2013) 504:451–5. doi: 10.1038/nature12726 24226773 PMC3869884

[38] Litvak Y Byndloss MX Baumler AJ . Colonocyte metabolism shapes the gut microbiota. Science. (2018) 362. doi: 10.1126/science.aat9076 30498100 PMC6296223

[39] Inagaki T Moschetta A Lee YK Peng L Zhao G Downes M . Regulation of antibacterial defense in the small intestine by the nuclear bile acid receptor. Proc Natl Acad Sci USA. (2006) 103:3920–5. doi: 10.1073/pnas.0509592103 16473946 PMC1450165

[40] Qin J Li R Raes J Arumugam M Burgdorf KS Manichanh C . A human gut microbial gene catalogue established by metagenomic sequencing. Nature. (2010) 464:59–65. doi: 10.1038/nature08821 20203603 PMC3779803

[41] Eckburg PB Bik EM Bernstein CN Purdom E Dethlefsen L Sargent M . Diversity of the human intestinal microbial flora. Science. (2005) 308:1635–8. doi: 10.1126/science.1110591 15831718 PMC1395357

[42] Arumugam M Raes J Pelletier E Le Paslier D Yamada T Mende DR . Enterotypes of the human gut microbiome. Nature. (2011) 473:174–80. doi: 10.1038/nature09944 21508958 PMC3728647

[43] Minot S Sinha R Chen J Li H Keilbaugh SA Wu GD . The human gut virome: inter-individual variation and dynamic response to diet. Genome Res. (2011) 21:1616–25. doi: 10.1101/gr.122705.111 21880779 PMC3202279

[44] Suhr MJ Banjara N Hallen-Adams HE . Sequence-based methods for detecting and evaluating the human gut mycobiome. Lett Appl Microbiol. (2016) 62:209–15. doi: 10.1111/lam.12539 26669281

[45] Demir M Lang S Hartmann P Duan Y Martin A Miyamoto Y . The fecal mycobiome in non-alcoholic fatty liver disease. J Hepatol. (2022) 76:788–99. doi: 10.1016/j.jhep.2021.11.029 34896404 PMC8981795

[46] Lang S Demir M Martin A Jiang L Zhang X Duan Y . Intestinal virome signature associated with severity of nonalcoholic fatty liver disease. Gastroenterology. (2020) 159:1839–52. doi: 10.1053/j.gastro.2020.07.005 32652145 PMC8404510

[47] Lang S Duan Y Liu J Torralba MG Kuelbs C Ventura-Cots M . Intestinal fungal dysbiosis and systemic immune response to fungi in patients with alcoholic hepatitis. Hepatology. (2020) 71:522–38. doi: 10.1002/hep.30832 31228214 PMC6925657

[48] Jiang L Lang S Duan Y Zhang X Gao B Chopyk J . Intestinal virome in patients with alcoholic hepatitis. Hepatology. (2020) 72:2182–96. doi: 10.1002/hep.31459 32654263 PMC8159727

[49] Bajaj JS Liu EJ Kheradman R Fagan A Heuman DM White M . Fungal dysbiosis in cirrhosis. Gut. (2018) 67:1146–54. doi: 10.1136/gutjnl-2016-313170 28578302

[50] Santus W Devlin JR Behnsen J . Crossing kingdoms: how the mycobiota and fungal-bacterial interactions impact host health and disease. Infect Immun. (2021) 89. doi: 10.1128/IAI.00648-20 33526565 PMC8090948

[51] Chu H Duan Y Lang S Jiang L Wang Y Llorente C . The candida albicans exotoxin candidalysin promotes alcohol-associated liver disease. J Hepatol. (2020) 72:391–400. doi: 10.1016/j.jhep.2019.09.029 31606552 PMC7031049

[52] Richardson JP Brown R Kichik N Lee S Priest E Mogavero S . Candidalysins are a new family of cytolytic fungal peptide toxins. mBio. (2022) 13:e0351021. doi: 10.1128/mbio.03510-21 35073742 PMC8787473

[53] Verma AH Richardson JP Zhou C Coleman BM Moyes DL Ho J . Oral epithelial cells orchestrate innate type 17 responses to candida albicans through the virulence factor candidalysin. Sci Immunol. (2017) 2. doi: 10.1126/sciimmunol.aam8834 29101209 PMC5881387

[54] Kasper L Konig A Koenig PA Gresnigt MS Westman J Drummond RA . The fungal peptide toxin candidalysin activates the Nlrp3 inflammasome and causes cytolysis in mononuclear phagocytes. Nat Commun. (2018) 9:4260. doi: 10.1038/s41467-018-06607-1 30323213 PMC6189146

[55] Duan Y Llorente C Lang S Brandl K Chu H Jiang L . Bacteriophage targeting of gut bacterium attenuates alcoholic liver disease. Nature. (2019) 575:505–11. doi: 10.1038/s41586-019-1742-x 31723265 PMC6872939

[56] Mazagova M Wang L Anfora AT Wissmueller M Lesley SA Miyamoto Y . Commensal microbiota is hepatoprotective and prevents liver fibrosis in mice. FASEB J. (2015) 29:1043–55. doi: 10.1096/fj.14-259515 25466902 PMC4422368

[57] Henao-Mejia J Elinav E Jin C Hao L Mehal WZ Strowig T . Inflammasome-mediated dysbiosis regulates progression of Nafld and obesity. Nature. (2012) 482:179–85. doi: 10.1038/nature10809 22297845 PMC3276682

[58] Llopis M Cassard AM Wrzosek L Boschat L Bruneau A Ferrere G . Intestinal microbiota contributes to individual susceptibility to alcoholic liver disease. Gut. (2016) 65:830–9. doi: 10.1136/gutjnl-2015-310585 26642859

[59] Fan Y Pedersen O . Gut microbiota in human metabolic health and disease. Nat Rev Microbiol. (2021) 19:55–71. doi: 10.1038/s41579-020-0433-9 32887946

[60] Friedman ES Li Y Shen TD Jiang J Chau L Adorini L . Fxr-dependent modulation of the human small intestinal microbiome by the bile acid derivative obeticholic acid. Gastroenterology. (2018) 155:1741–52:e5. doi: 10.1053/j.gastro.2018.08.022 30144429 PMC6279623

[61] Kakiyama G Pandak WM Gillevet PM Hylemon PB Heuman DM Daita K . Modulation of the fecal bile acid profile by gut microbiota in cirrhosis. J Hepatol. (2013) 58:949–55. doi: 10.1016/j.jhep.2013.01.003 23333527 PMC3936319

[62] Fukui H Wiest R . Changes of intestinal functions in liver cirrhosis. Inflammation Intest Dis. (2016) 1:24–40. doi: 10.1159/000444436 29922655 PMC5988129

[63] Brandl K Hartmann P Jih LJ Pizzo DP Argemi J Ventura-Cots M . Dysregulation of serum bile acids and Fgf19 in alcoholic hepatitis. J Hepatol. (2018) 69:396–405. doi: 10.1016/j.jhep.2018.03.031 29654817 PMC6054564

[64] Hasa E Hartmann P Schnabl B . Liver cirrhosis and immune dysfunction. Int Immunol. (2022) 34:455–66. doi: 10.1093/intimm/dxac030 35792761 PMC9447994

[65] Kurioka A Walker LJ Klenerman P Willberg CB . Mait cells: New guardians of the liver. Clin Transl Immunol. (2016) 5:e98. doi: 10.1038/cti.2016.51 27588203 PMC5007630

[66] Riva A Patel V Kurioka A Jeffery HC Wright G Tarff S . Mucosa-associated invariant T cells link intestinal immunity with antibacterial immune defects in alcoholic liver disease. Gut. (2018) 67:918–30. doi: 10.1136/gutjnl-2017-314458 29097439 PMC5890654

[67] Hegde P Weiss E Paradis V Wan J Mabire M Sukriti S . Mucosal-associated invariant T cells are a profibrogenic immune cell population in the liver. Nat Commun. (2018) 9:2146. doi: 10.1038/s41467-018-04450-y 29858567 PMC5984626

[68] Zhang Y Kong D Wang H . Mucosal-associated invariant T cell in liver diseases. Int J Biol Sci. (2020) 16:460–70. doi: 10.7150/ijbs.39016 32015682 PMC6990906

[69] Li Y Huang B Jiang X Chen W Zhang J Wei Y . Mucosal-associated invariant T cells improve nonalcoholic fatty liver disease through regulating macrophage polarization. Front Immunol. (2018) 9:1994. doi: 10.3389/fimmu.2018.01994 30233587 PMC6131560

[70] Ma C Han M Heinrich B Fu Q Zhang Q Sandhu M . Gut microbiome-mediated bile acid metabolism regulates liver cancer via Nkt cells. Science. (2018) 360. doi: 10.1126/science.aan5931 29798856 PMC6407885

[71] Kulik L El-Serag HB . Epidemiology and management of hepatocellular carcinoma. Gastroenterology. (2019) 156:477–91:e1. doi: 10.1053/j.gastro.2018.08.065 30367835 PMC6340716

[72] Campbell C McKenney PT Konstantinovsky D Isaeva OI Schizas M Verter J . Bacterial metabolism of bile acids promotes generation of peripheral regulatory T cells. Nature. (2020) 581:475–9. doi: 10.1038/s41586-020-2193-0 32461639 PMC7540721

[73] Song X Sun X Oh SF Wu M Zhang Y Zheng W . Microbial bile acid metabolites modulate gut Rorgamma(+) regulatory T cell homeostasis. Nature. (2020) 577:410–5. doi: 10.1038/s41586-019-1865-0 31875848 PMC7274525

[74] Hang S Paik D Yao L Kim E Trinath J Lu J . Bile acid metabolites control T(H)17 and T(Reg) cell differentiation. Nature. (2019) 576:143–8. doi: 10.1038/s41586-019-1785-z 31776512 PMC6949019

[75] Teunis C Nieuwdorp M Hanssen N . Interactions between tryptophan metabolism, the gut microbiome and the immune system as potential drivers of non-alcoholic fatty liver disease (Nafld) and metabolic diseases. Metabolites. (2022) 12. doi: 10.3390/metabo12060514 35736447 PMC9227929

[76] Jiao N Baker SS Chapa-Rodriguez A Liu W Nugent CA Tsompana M . Suppressed hepatic bile acid signalling despite elevated production of primary and secondary bile acids in Nafld. Gut. (2018) 67:1881–91. doi: 10.1136/gutjnl-2017-314307 28774887

[77] Ferslew BC Xie G Johnston CK Su M Stewart PW Jia W . Altered bile acid metabolome in patients with nonalcoholic steatohepatitis. Dig Dis Sci. (2015) 60:3318–28. doi: 10.1007/s10620-015-3776-8 26138654 PMC4864493

[78] Wrzosek L Ciocan D Hugot C Spatz M Dupeux M Houron C . Microbiota tryptophan metabolism induces aryl hydrocarbon receptor activation and improves alcohol-induced liver injury. Gut. (2021) 70:1299–308. doi: 10.1136/gutjnl-2020-321565 33004548

[79] Grant AJ Lalor PF Salmi M Jalkanen S Adams DH . Homing of mucosal lymphocytes to the liver in the pathogenesis of hepatic complications of inflammatory bowel disease. Lancet. (2002) 359:150–7. doi: 10.1016/S0140-6736(02)07374-9 11809275

[80] Eksteen B Grant AJ Miles A Curbishley SM Lalor PF Hubscher SG . Hepatic endothelial Ccl25 mediates the recruitment of Ccr9+ gut-homing lymphocytes to the liver in primary sclerosing cholangitis. J Exp Med. (2004) 200:1511–7. doi: 10.1084/jem.20041035 15557349 PMC2211943

[81] Chaudhry S Emond J Griesemer A . Immune cell trafficking to the liver. Transplantation. (2019) 103:1323–37. doi: 10.1097/TP.0000000000002690 30817405 PMC7044802

[82] de Krijger M Visseren T Wildenberg ME Hooijer GKJ Verstegen MMA van der Laan LJW . Characterization of gut-homing molecules in non-endstage livers of patients with primary sclerosing cholangitis and inflammatory bowel disease. J Transl Autoimmun. (2020) 3:100054. doi: 10.1016/j.jtauto.2020.100054 32743534 PMC7388383

[83] Ramachandran P Dobie R Wilson-Kanamori JR Dora EF Henderson BEP Luu NT . Resolving the fibrotic niche of human liver cirrhosis at single-cell level. Nature. (2019) 575:512–8. doi: 10.1038/s41586-019-1631-3 31597160 PMC6876711

[84] Turner JR . Intestinal mucosal barrier function in health and disease. Nat Rev Immunol. (2009) 9:799–809. doi: 10.1038/nri2653 19855405

[85] Rahman K Desai C Iyer SS Thorn NE Kumar P Liu Y . Loss of junctional adhesion molecule a promotes severe steatohepatitis in mice on a diet high in saturated fat, fructose, and cholesterol. Gastroenterology. (2016) 151:733–46:e12. doi: 10.1053/j.gastro.2016.06.022 27342212 PMC5037035

[86] Luther J Garber JJ Khalili H Dave M Bale SS Jindal R . Hepatic injury in nonalcoholic steatohepatitis contributes to altered intestinal permeability. Cell Mol Gastroenterol Hepatol. (2015) 1:222–32. doi: 10.1016/j.jcmgh.2015.01.001 26405687 PMC4578658

[87] Bauer TM Schwacha H Steinbruckner B Brinkmann FE Ditzen AK Aponte JJ . Small intestinal bacterial overgrowth in human cirrhosis is associated with systemic endotoxemia. Am J Gastroenterol. (2002) 97:2364–70. doi: 10.1111/j.1572-0241.2002.05791.x 12358257

[88] Chang CS Chen GH Lien HC Yeh HZ . Small intestine dysmotility and bacterial overgrowth in cirrhotic patients with spontaneous bacterial peritonitis. Hepatology. (1998) 28:1187–90. doi: 10.1002/hep.510280504 9794900

[89] Dapito DH Mencin A Gwak GY Pradere JP Jang MK Mederacke I . Promotion of hepatocellular carcinoma by the intestinal microbiota and Tlr4. Cancer Cell. (2012) 21:504–16. doi: 10.1016/j.ccr.2012.02.007 22516259 PMC3332000

[90] Yu LX Yan HX Liu Q Yang W Wu HP Dong W . Endotoxin accumulation prevents carcinogen-induced apoptosis and promotes liver tumorigenesis in rodents. Hepatology. (2010) 52:1322–33. doi: 10.1002/hep.23845 20803560

[91] Mouzaki M Wang AY Bandsma R Comelli EM Arendt BM Zhang L . Bile acids and dysbiosis in non-alcoholic fatty liver disease. PloS One. (2016) 11:e0151829. doi: 10.1371/journal.pone.0151829 27203081 PMC4874546

[92] Hartmann P Hochrath K Horvath A Chen P Seebauer CT Llorente C . Modulation of the intestinal bile acid/farnesoid X receptor/fibroblast growth factor 15 axis improves alcoholic liver disease in mice. Hepatology. (2018) 67:2150–66. doi: 10.1002/hep.29676 29159825 PMC5962369

[93] Morrison DJ Preston T . Formation of short chain fatty acids by the gut microbiota and their impact on human metabolism. Gut Microbes. (2016) 7:189–200. doi: 10.1080/19490976.2015.1134082 26963409 PMC4939913

[94] Beaumont M Neyrinck AM Olivares M Rodriguez J de Rocca Serra A Roumain M . The gut microbiota metabolite indole alleviates liver inflammation in mice. FASEB J. (2018) 32:fj201800544. doi: 10.1096/fj.201800544 29906245 PMC6219839

[95] Ma L Li H Hu J Zheng J Zhou J Botchlett R . Indole alleviates diet-induced hepatic steatosis and inflammation in a manner involving myeloid cell 6-phosphofructo-2-kinase/fructose-2,6-biphosphatase 3. Hepatology. (2020) 72:1191–203. doi: 10.1002/hep.31115 31953865 PMC7365739

[96] Meijnikman AS Davids M Herrema H Aydin O Tremaroli V Rios-Morales M . Microbiome-derived ethanol in nonalcoholic fatty liver disease. Nat Med. (2022) 28:2100–6. doi: 10.1038/s41591-022-02016-6 36216942

[97] Zhu L Baker SS Gill C Liu W Alkhouri R Baker RD . Characterization of gut microbiomes in nonalcoholic steatohepatitis (Nash) patients: A connection between endogenous alcohol and Nash. Hepatology. (2013) 57:601–9. doi: 10.1002/hep.26093 23055155

[98] Yuan J Chen C Cui J Lu J Yan C Wei X . Fatty liver disease caused by high-alcohol-producing Klebsiella pneumoniae. Cell Metab. (2019) 30:675–88:e7. doi: 10.1016/j.cmet.2019.08.018 31543403

[99] Helsley RN Miyata T Kadam A Varadharajan V Sangwan N Huang EC . Gut microbial trimethylamine is elevated in alcohol-associated hepatitis and contributes to ethanol-induced liver injury in mice. Elife. (2022) 11. doi: 10.7554/eLife.76554 35084335 PMC8853661

[100] Sayin SI Wahlstrom A Felin J Jantti S Marschall HU Bamberg K . Gut microbiota regulates bile acid metabolism by reducing the levels of tauro-beta-muricholic acid, a naturally occurring Fxr antagonist. Cell Metab. (2013) 17:225–35. doi: 10.1016/j.cmet.2013.01.003 23395169

[101] Powell EE Wong VW Rinella M . Non-alcoholic fatty liver disease. Lancet. (2021) 397:2212–24. doi: 10.1016/S0140-6736(20)32511-3 33894145

[102] Loomba R Seguritan V Li W Long T Klitgord N Bhatt A . Gut microbiome-based metagenomic signature for non-invasive detection of advanced fibrosis in human nonalcoholic fatty liver disease. Cell Metab. (2017) 25:1054–62:e5. doi: 10.1016/j.cmet.2017.04.001 28467925 PMC5502730

[103] Lee G You HJ Bajaj JS Joo SK Yu J Park S . Distinct signatures of gut microbiome and metabolites associated with significant fibrosis in non-obese NAFLD. Nat Commun. (2020) 11:4982. doi: 10.1038/s41467-020-18754-5 33020474 PMC7536225

[104] Fang S Suh JM Reilly SM Yu E Osborn O Lackey D . Intestinal FXR agonism promotes adipose tissue browning and reduces obesity and insulin resistance. Nat Med. (2015) 21:159–65. doi: 10.1038/nm.3760 25559344 PMC4320010

[105] Jiang C Xie C Li F Zhang L Nichols RG Krausz KW . Intestinal farnesoid X receptor signaling promotes nonalcoholic fatty liver disease. J Clin Invest. (2015) 125:386–402. doi: 10.1172/JCI76738 25500885 PMC4382255

[106] Leclercq S Matamoros S Cani PD Neyrinck AM Jamar F Starkel P . Intestinal permeability, gut-bacterial dysbiosis, and behavioral markers of alcohol-dependence severity. Proc Natl Acad Sci USA. (2014) 111:E4485–93. doi: 10.1073/pnas.1415174111 25288760 PMC4210345

[107] Wang L Fouts DE Starkel P Hartmann P Chen P Llorente C . Intestinal Reg3 lectins protect against alcoholic steatohepatitis by reducing mucosa-associated microbiota and preventing bacterial translocation. Cell Host Microbe. (2016) 19:227–39. doi: 10.1016/j.chom.2016.01.003 26867181 PMC4786170

[108] Hsu CL Zhang X Jiang L Lang S Hartmann P Pride D . Intestinal virome in patients with alcohol use disorder and after abstinence. Hepatol Commun. (2022) 6:2058–69. doi: 10.1002/hep4.1947 35368152 PMC9315129

[109] Cresci GA Glueck B McMullen MR Xin W Allende D Nagy LE . Prophylactic tributyrin treatment mitigates chronic-binge ethanol-induced intestinal barrier and liver injury. J Gastroenterol Hepatol. (2017) 32:1587–97. doi: 10.1111/jgh.13731 28087985 PMC5511097

[110] Hendrikx T Duan Y Wang Y Oh JH Alexander LM Huang W . Bacteria engineered to produce IL-22 in intestine induce expression of Reg3g to reduce ethanol-induced liver disease in mice. Gut. (2019) 68:1504–15. doi: 10.1136/gutjnl-2018-317232 30448775 PMC6387784

[111] Grander C Adolph TE Wieser V Lowe P Wrzosek L Gyongyosi B . Recovery of ethanol-induced Akkermansia muciniphila depletion ameliorates alcoholic liver disease. Gut. (2018) 67:891–901. doi: 10.1136/gutjnl-2016-313432 28550049

[112] Bajaj JS Sikaroodi M Shamsaddini A Henseler Z Santiago-Rodriguez T Acharya C . Interaction of bacterial metagenome and virome in patients with cirrhosis and hepatic encephalopathy. Gut. (2021) 70:1162–73. doi: 10.1136/gutjnl-2020-322470 32998876

[113] Bajaj JS . Alcohol, liver disease and the gut microbiota. Nat Rev Gastroenterol Hepatol. (2019) 16:235–46. doi: 10.1038/s41575-018-0099-1 30643227

[114] Bajaj JS Kakiyama G Savidge T Takei H Kassam ZA Fagan A . Antibiotic-associated disruption of microbiota composition and function in cirrhosis is restored by fecal transplant. Hepatology. (2018) 68:1549–58. doi: 10.1002/hep.30037 29665102

[115] Bajaj JS Salzman NH Acharya C Sterling RK White MB Gavis EA . Fecal microbial transplant capsules are safe in hepatic encephalopathy: a phase 1, randomized, placebo-controlled trial. Hepatology. (2019) 70:1690–703. doi: 10.1002/hep.30690 31038755 PMC6819208

[116] Ianiro G Puncochar M Karcher N Porcari S Armanini F Asnicar F . Variability of strain engraftment and predictability of microbiome composition after fecal microbiota transplantation across different diseases. Nat Med. (2022) 28:1913–23. doi: 10.1038/s41591-022-01964-3 36109637 PMC9499858

[117] Ng SC Xu Z Mak JWY Yang K Liu Q Zuo T . Microbiota engraftment after faecal microbiota transplantation in obese subjects with type 2 diabetes: a 24-week, double-blind, randomised controlled trial. Gut. (2022) 71:716–23. doi: 10.1136/gutjnl-2020-323617 33785557

[118] DeFilipp Z Bloom PP Torres Soto M Mansour MK Sater MRA Huntley MH . Drug-resistant E. coli bacteremia transmitted by fecal microbiota transplant. N Engl J Med. (2019) 381:2043–50. doi: 10.1056/NEJMoa1910437 31665575

[119] Ren Z Li A Jiang J Zhou L Yu Z Lu H . Gut microbiome analysis as a tool towards targeted non-invasive biomarkers for early hepatocellular carcinoma. Gut. (2019) 68:1014–23. doi: 10.1136/gutjnl-2017-315084 30045880 PMC6580753

[120] Ponziani FR Bhoori S Castelli C Putignani L Rivoltini L Del Chierico F . Hepatocellular carcinoma is associated with gut microbiota profile and inflammation in nonalcoholic fatty liver disease. Hepatology. (2019) 69:107–20. doi: 10.1002/hep.30036 29665135

[121] Zheng R Wang G Pang Z Ran N Gu Y Guan X . Liver cirrhosis contributes to the disorder of gut microbiota in patients with hepatocellular carcinoma. Cancer Med. (2020) 9:4232–50. doi: 10.1002/cam4.3045 32281295 PMC7300425

[122] Yoshimoto S Loo TM Atarashi K Kanda H Sato S Oyadomari S . Obesity-induced gut microbial metabolite promotes liver cancer through senescence secretome. Nature. (2013) 499:97–101. doi: 10.1038/nature12347 23803760

[123] Iida N Mizukoshi E Yamashita T Yutani M Seishima J Wang Z . Chronic liver disease enables gut Enterococcus faecalis colonization to promote liver carcinogenesis. Nat Cancer. (2021) 2:1039–54. doi: 10.1038/s43018-021-00251-3 35121877

[124] Yang Y Nguyen M Khetrapal V Sonnert ND Martin AL Chen H . Within-host evolution of a gut pathobiont facilitates liver translocation. Nature. (2022) 607:563–70. doi: 10.1038/s41586-022-04949-x 35831502 PMC9308686

[125] Schwabe RF Greten TF . Gut microbiome in HCC - mechanisms, diagnosis and therapy. J Hepatol. (2020) 72:230–38. doi: 10.1016/j.jhep.2019.08.016 31954488

[126] Hsu CL Duan Y Fouts DE Schnabl B . Intestinal virome and therapeutic potential of bacteriophages in liver disease. J Hepatol. (2021) 75:1465–75. doi: 10.1016/j.jhep.2021.08.003 34437908 PMC8929164

[127] Kurtz CB Millet YA Puurunen MK Perreault M Charbonneau MR Isabella VM . An engineered E. coli Nissle improves hyperammonemia and survival in mice and shows dose-dependent exposure in healthy humans. Sci Transl Med. (2019) 11. doi: 10.1126/scitranslmed.aau7975 30651324

[128] Russell BJ Brown SD Siguenza N Mai I Saran AR Lingaraju A . Intestinal transgene delivery with native E. coli chassis allows persistent physiological changes. Cell. (2022) 185:3263–77:e15. doi: 10.1016/j.cell.2022.06.050 35931082 PMC9464905

[129] Younossi ZM Ratziu V Loomba R Rinella M Anstee QM Goodman Z . Obeticholic acid for the treatment of non-alcoholic steatohepatitis: interim analysis from a multicentre, randomised, placebo-controlled phase 3 trial. Lancet. (2019) 394:2184–96. doi: 10.1016/S0140-6736(19)33041-7 31813633

[130] Harrison SA Bedossa P Guy CD Schattenberg JM Loomba R Taub R . A phase 3, randomized, controlled trial of resmetirom in NASH with liver fibrosis. N Engl J Med. (2024) 390:497–509. doi: 10.1056/NEJMoa2309000 38324483

[131] Philips CA Ahamed R Rajesh S Abduljaleel JKP Augustine P . Long-term outcomes of stool transplant in alcohol-associated hepatitis-analysis of clinical outcomes, relapse, gut microbiota and comparisons with standard care. J Clin Exp Hepatol. (2022) 12:1124–32. doi: 10.1016/j.jceh.2022.01.001 35814513 PMC9257856

[132] Bajaj JS Gavis EA Fagan A Wade JB Thacker LR Fuchs M . A randomized clinical trial of fecal microbiota transplant for alcohol use disorder. Hepatology. (2021) 73:1688–700. doi: 10.1002/hep.31496 32750174

[133] Friedman SL Ratziu V Harrison SA Abdelmalek MF Aithal GP Caballeria J . A randomized, placebo-controlled trial of cenicriviroc for treatment of nonalcoholic steatohepatitis with fibrosis. Hepatology. (2018) 67:1754–67. doi: 10.1002/hep.29477 28833331 PMC5947654

[134] Lynch KD Chapman RW Keshav S Montano-Loza AJ Mason AL Kremer AE . Effects of vedolizumab in patients with primary sclerosing cholangitis and inflammatory bowel diseases. Clin Gastroenterol Hepatol. (2020) 18:179–87:e6. doi: 10.1016/j.cgh.2019.05.013 31100458 PMC6941216

[135] Trapecar M Communal C Velazquez J Maass CA Huang YJ Schneider K . Gut-liver physiomimetics reveal paradoxical modulation of IBD-related inflammation by short-chain fatty acids. Cell Syst. (2020) 10:223–39:e9. doi: 10.1016/j.cels.2020.02.008 32191873 PMC8143761

[136] Andrews TS Nakib D Perciani CT Ma XZ Liu L Winter E . Single-cell, single-nucleus, and spatial transcriptomics characterization of the immunological landscape in the healthy and PSC human liver. J Hepatol. (2024) 80:730–43. doi: 10.1016/j.jhep.2023.12.023 38199298

[137] Watson BR Paul B Rahman RU Amir-Zilberstein L Segerstolpe A Epstein ET . Spatial transcriptomics of healthy and fibrotic human liver at single-cell resolution. Nat Commun. (2025) 16:319. doi: 10.1038/s41467-024-55325-4 39747812 PMC11697218

[138] Ben-Moshe S Itzkovitz S . Spatial heterogeneity in the mammalian liver. Nat Rev Gastroenterol Hepatol. (2019) 16:395–410. doi: 10.1038/s41575-019-0134-x 30936469

[139] Halpern KB Shenhav R Matcovitch-Natan O Toth B Lemze D Golan M . Single-cell spatial reconstruction reveals global division of labour in the mammalian liver. Nature. (2017) 542:352–56. doi: 10.1038/nature21065 28166538 PMC5321580

[140] Paris J Henderson NC . Liver zonation, revisited. Hepatology. (2022) 76:1219–30. doi: 10.1002/hep.32408 35175659 PMC9790419

[141] Walter J Armet AM Finlay BB Shanahan F . Establishing or exaggerating causality for the gut microbiome: lessons from human microbiota-associated rodents. Cell. (2020) 180:221–32. doi: 10.1016/j.cell.2019.12.025 31978342

[142] Molinaro A Wahlstrom A Marschall HU . Role of bile acids in metabolic control. Trends Endocrinol Metab. (2018) 29:31–41. doi: 10.1016/j.tem.2017.11.002 29195686

[143] Mowat AM Agace WW . Regional specialization within the intestinal immune system. Nat Rev Immunol. (2014) 14:667–85. doi: 10.1038/nri3738 25234148

[144] Sharma SP Gupta H Kwon GH Lee SY Song SH Kim JS . Gut microbiome and metabolome signatures in liver cirrhosis-related complications. Clin Mol Hepatol. (2024) 30:845–62. doi: 10.3350/cmh.2024.0349 39048520 PMC11540350

[145] Sharpton SR Oh TG Madamba E Wang C Yu RT Atkins AR . Gut metagenome-derived signature predicts hepatic decompensation and mortality in NAFLD-related cirrhosis. Aliment Pharmacol Ther. (2022) 56:1475–85. doi: 10.1111/apt.17236 36164267 PMC9746351

[146] Oh TG Kim SM Caussy C Fu T Guo J Bassirian S . A universal gut-microbiome-derived signature predicts cirrhosis. Cell Metab. (2020) 32:878–88:e6. doi: 10.1016/j.cmet.2020.06.005 32610095 PMC7822714

