# Correction: The gut-liver-immune axis: structural basis, cellular architecture, molecular mediators, and disease-specific manifestations

**DOI:** 10.3389/fimmu.2026.1960070

**Published:** 2026-08-14

**Authors:** Sang Jun Yoon, Jung A Eom, Ki Tae Suk

**Affiliations:** Institute for Liver and Digestive Diseases, Hallym University College of Medicine, Chuncheon, Republic of Korea

**Keywords:** bile acids, gut-liver axis, gut-liver-immune axis, intestinal barrier, liver disease, macrophages, MAIT cells, Treg/Th17 balance

Author Jung A Eom was erroneously omitted as equal contributing author. The original version of this article has been updated.

